# Esc peptides and derivatives potentiate the activity of CFTR with gating defects and display antipseudomonal activity in cystic fibrosis-like lung disease

**DOI:** 10.1007/s00018-025-05633-9

**Published:** 2025-03-18

**Authors:** Loretta Ferrera, Floriana Cappiello, Arianna Venturini, Hexin Lu, Bruno Casciaro, Giacomo Cappella, Giulio Bontempi, Alessandra Corrente, Raffaele Strippoli, Federico Zara, Y. Peter Di, Luis J. V. Galietta, Mattia Mori, Maria Luisa Mangoni

**Affiliations:** 1 UOC Genetica Medica, IRCCS Istituto Giannina Gaslini, Genoa, Italy; 2 Laboratory Affiliated to Pasteur Italia-Fondazione Cenci Bolognetti, Department of Biochemical Sciences, Sapienza University of Rome, Rome, Italy; 3 Telethon Institute of Genetics and Medicine (TIGEM), Pozzuoli, Naples Italy; 4 Department of Environmental and Occupational Health, University of Pittsburgh, Pittsburgh, USA; 5 Department of Molecular Medicine, Sapienza University of Rome, Rome, Italy; 6 National Institute for Infectious Diseases L. Spallanzani IRCCS, Via Portuense, 292, 00149 Rome, Italy; 7 Department of Neurosciences, Rehabilitation, Ophthalmology, Genetics, Maternal and Child Health (Dinogmi), University of Genoa, Genoa, Italy; 8 Department of Translational Medical Sciences, University of Napoli “Federico II”, Naples, Italy; 9 Department of Biotechnology, Chemistry and Pharmacy, University of Siena, Siena, Italy

**Keywords:** Antimicrobial peptides, CFTR potentiators, Cystic fibrosis, Antibiotic resistance, Lung infection

## Abstract

Cystic fibrosis (CF) is a rare disease caused by mutations in the gene encoding the CF transmembrane conductance regulator (CFTR), a chloride channel with an important role in the airways. Despite the clinical efficacy of present modulators in restoring the activity of defective CFTR, there are patients who show persistent pulmonary infections, mainly due to *Pseudomonas aeruginosa*. Recently, we reported an unprecedented property of antimicrobial peptides i.e. Esc peptides, which consists in their ability to act as potentiators of CFTR carrying the most common mutation (the loss of phenylalanine 508) affecting protein folding, trafficking and gating. In this work, by electrophysiology experiments and computational studies, the capability of these peptides and de-novo designed analogs was demonstrated to recover the function of other mutated forms of CFTR which severely affect the channel gating (G551D and G1349D). This is presumably due to direct interaction of the peptides with the nucleotide binding domains (NBDs) of CFTR, followed by a novel local phenomenon consisting in distancing residues located at the cytosolic side of the NBDs interface, thus stabilizing the open conformation of the pore at its cytosolic end. The most promising peptides for the dual antimicrobial and CFTR potentiator activities were also shown to display antipseudomonal activity in conditions mimicking the CF pulmonary ion transport and mucus obstruction, with a higher efficacy than the clinically used colistin. These studies should assist in development of novel drugs for lung pathology in CF, with dual CFTR potentiator and large spectrum antibiotic activities.

**Supplementary Information:**

The online version contains supplementary material available at 10.1007/s00018-025-05633-9.

## Introduction

Cystic fibrosis (CF) is a genetic disorder due to mutations in the gene encoding the CF transmembrane conductance regulator (CFTR), a kinase-activated and ATP-gated channel protein controlling the transport of chloride (Cl^−^) and bicarbonate (HCO_3_^-^) anions across mucosal surfaces, including those at the airways, and hence, the composition of the periciliary airway liquid [1–4]. CFTR contains two transmembrane domains (TMD1 and TMD2), two nucleotide binding domains (NBD1 and NBD2), and a regulatory R domain [5]. To allow the passage of ions, CFTR needs to be phosphorylated at multiple sites of the R portion [6], by the cAMP-dependent protein kinase A (pKA) and to bind two ATP molecules at the NBDs, with subsequent NBD dimerization and opening of the transmembrane channel [7–9]. So far, more than two thousand mutations have been identified in the CFTR gene, from a complete absence of the protein to its partial dysfunction [10], and categorized into 6 different classes [11], while about 380 mutations have been verified as pathogenic [9, 12–14]. The most prevalent genetic variant in CF is the loss of phenylalanine 508 in the NBD1 domain of CFTR (F508del-CFTR). This compromises CFTR trafficking to the plasma membrane and interferes with the mechanisms of channel opening [15, 16]. This mutation belongs to class II and affects around 90% of people with CF carrying at least one copy of this altered gene [17]. For missense mutations belonging to class III, such as G551D or G1349D, in which glycine is replaced by aspartic acid at two different positions of the “signature sequence” essential for ATP binding (551 in the NBD1 and 1349 in the NBD2, respectively) [18], the mutated protein correctly reaches the plasma membrane, but its gating is severely impaired. Irrespective of their mechanism of action, CF mutations inhibit transepithelial anion transport in epithelial cells, with serious consequences in the airways. Hence, defective anion transport causes dehydration of airway surface and the formation of a sticky mucus coating the epithelial cells [2, 19, 20]. Such an event favours the accumulation and entrapment of inhaled microorganisms, including the opportunistic Gram-negative bacterium *Pseudomonas aeruginosa,* which lives everywhere, especially in moist environments like hospitals [21]. *P. aeruginosa* rapidly colonizes the lungs, forming biofilms that strongly resist the activity of traditional antibiotics, and induces a chronic infection with deterioration of pulmonary tissue and failure of respiratory function [22, 23].

The research community working on CF is trying to identify small compounds that can act as CFTR modulators, by assisting the delivery of the mutated channel to the plasma membrane (namely correctors) and/or by increasing the ions transport through the plasma membrane (namely potentiators) [24]. The combination of the corrector elexacaftor (VX-445), tezacaftor (VX-661) and the potentiator ivacaftor (VX-770) was successfully approved for therapy of CF patients with at least one allele of the most common F508del-CFTR mutation [25]. However, despite the clinical efficacy of current CFTR modulators in restoring the activity of defective CFTR [26], some patients cannot be treated or show persistent pulmonary infections (mainly due to *P. aeruginosa*) that remain a major challenge, mostly in advanced stages of lung disease [27]. Furthermore, the potentiator VX-770 only partially restores the activity of G551D-CFTR channel and patients with this defect still experience progressive impairment of lung function [28]. Based on these findings, an ideal treatment for lung pathology in CF may benefit from a drug having more than a single biological function.

In the last years, we identified two antimicrobial peptides (AMPs), Esculentin-1a(1–21)NH_2_ [Esc(1–21)] and its diastereomer Esc(1–21)−1c (Esc peptides, Table 1), with a strong activity against the free-living and sessile forms of *P. aeruginosa* and with the ability to accelerate recovery of damaged bronchial epithelium [29]. In addition, Esc(1–21)−1c was found to reduce lung bacterial burden in C57BL/6 mice models of acute *P. aeruginosa* lung infection, upon intratracheal (i.t.) instillation at 0.1 mg/Kg (corresponding to 20 μM) [30], especially when incorporated into biodegradable polymeric poly (lactic-co-glycolic acid) nanoparticles [31, 32]. Very recently, an unprecedented property of AMPs was reported, that is the ability of Esc peptides and the isoform d-phosphoEsc carrying a d-leucine at position 14 and d-phosphoserine at position 17 (Table 1), to act as potentiators of F508del-CFTR [33], likely upon direct interaction with the cytosolic NBD domains.

**Table 1.**
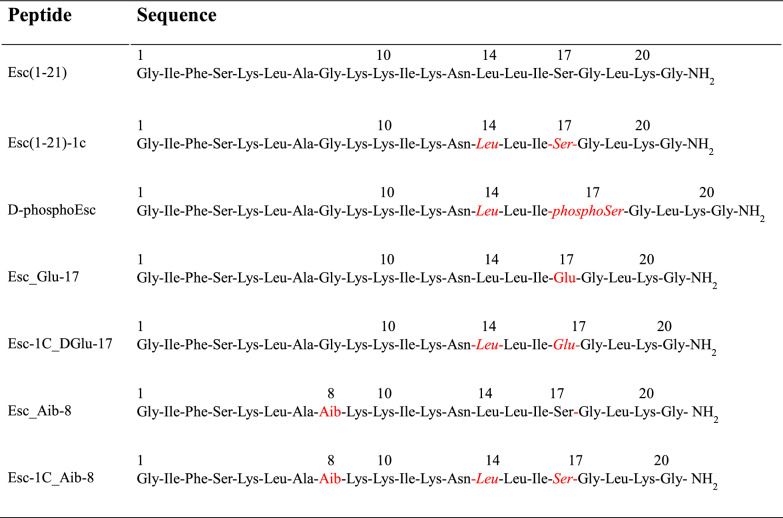
Primary structure of analogs of Esc peptides

^†^Amino acids substitution compared to Esc(1–21) are indicated in red. Amino acids in d configuration are in italics

Considering the high potentiality of Esc peptides to work as novel therapeutics, the objectives of the present study aimed at i) designing new Esc peptide analogs to optimize their efficacy for the dual antimicrobial and CFTR potentiator function; ii) investigating their effect on the ion currents mediated by different pathogenic variants of CFTR along with the underlying molecular mechanism; and iii) evaluating the capability of selected peptides to preserve antibacterial activity in CF-mimicking lung disease.

For the achievement of these objectives, electrophysiology experiments, molecular dynamic simulation and in vivo studies on proper mouse models were carried out.

## Results

### Design of new analogs of Esc peptides

Taking into account the higher efficacy of d-phosphoEsc peptide (Table 1) in rescuing F508del-CFTR activity in Fisher Rat Thyroid cells (FRT) [33], we investigated whether this outcome was ascribed to the presence of a negatively charged residue at position 17; therefore, two analogs containing d or l- Glutamic acid (Glu)^17^ were synthesized (Table 1). Furthermore, an analog bearing the non-coded alfa-aminoisobutyric acid (Aib) at position 8, instead of the achiral glycine i.e. [Aib^8^]-Esc(1–21), designated as Esc_Aib-8, and lately found to possess a larger spectrum of activity, being active also towards Gram-positive bacteria [34], was included for comparison, together with its diastereomer containing d-Leu^14^ and d-Ser^17^ (Table 1). All these new peptide isoforms were then analyzed for their effect on F508del-CFTR.

### Effect of peptides on FRT cells expressing F508del-CFTR

**Fig. 1:**
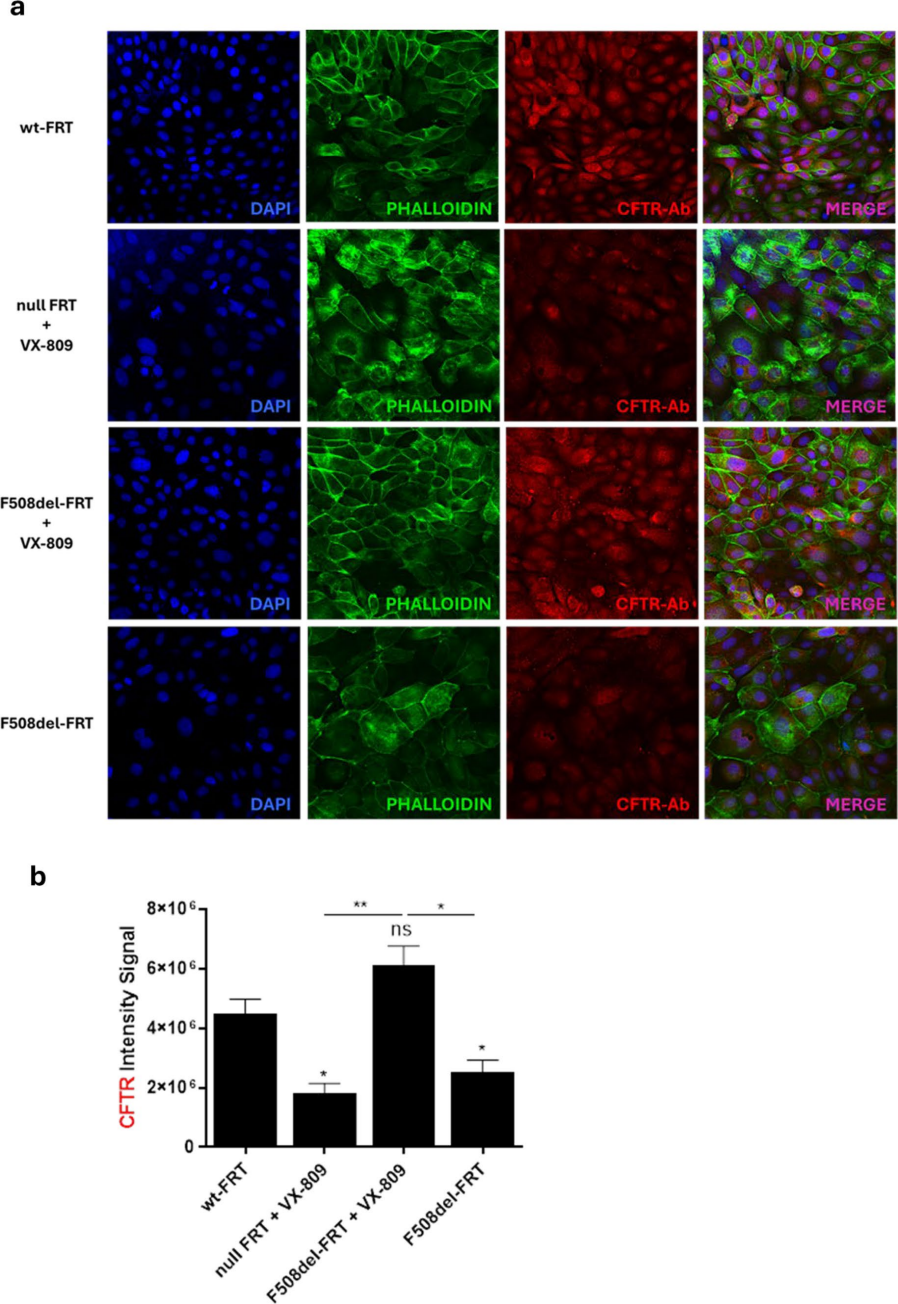
**a**. Representative confocal images of immunofluorescence to evaluate CFTR expression in wild type wt-FRT, null FRT and F508del-FRT cells with or without 3 μM VX-809 treatment, as indicated on the left side. Cells were stained with CFTR antibody (CFTR-Ab), 4′,6-diamidino-2-phenylindole (DAPI) and phalloidin as described in Materials and Methods. Scale bars, 10 μm. **b.** Quantitative evaluation of CFTR expression. The level of statistical significance of samples *versus* wt-FRT is indicated as follows: * p < 0.05. ns, not significant. The level of statistical significance between groups is also reported ** p < 0.01

The new de-novo designed peptides were initially used for transepithelial electrical resistance (TEER) experiments in FRT cells expressing F508del-CFTR (F508del-FRT) and pretreated with lumacaftor (VX-809) to assist the delivery of the mutated protein to the plasma membrane. The expression of CFTR in FRT cells was confirmed by confocal microscopy using anti-CFTR antibody for immunofluorescence studies. As reported in Fig. 1, a clear CFTR staining was observed in FRT cells expressing the wild-type protein (wt-FRT) or the mutated F508del-CFTR after treatment with VX-809 (to prevent the early degradation of the mutated protein and thus promoting its transport towards the cell membrane), compared to the results obtained in F508del-FRT cells that were not corrected with VX-809 or in cells that do not express CFTR (nullFRT).

To find out whether the selected peptides were able to improve the ions transport ability of F508del-CFTR, they were tested in F508del-FRT epithelium, in combination with forskolin (FSK), and the results were compared to those obtained by the application of FSK alone or FSK plus genistein (GEN) that was used as positive control. Indeed, FSK promotes CFTR phosphorylation by increasing the intracellular level of cAMP [35], whereas GEN is a known CFTR potentiator that accelerates channel opening and slows channel closure by direct interaction with the channel [36].

**Fig. 2:**
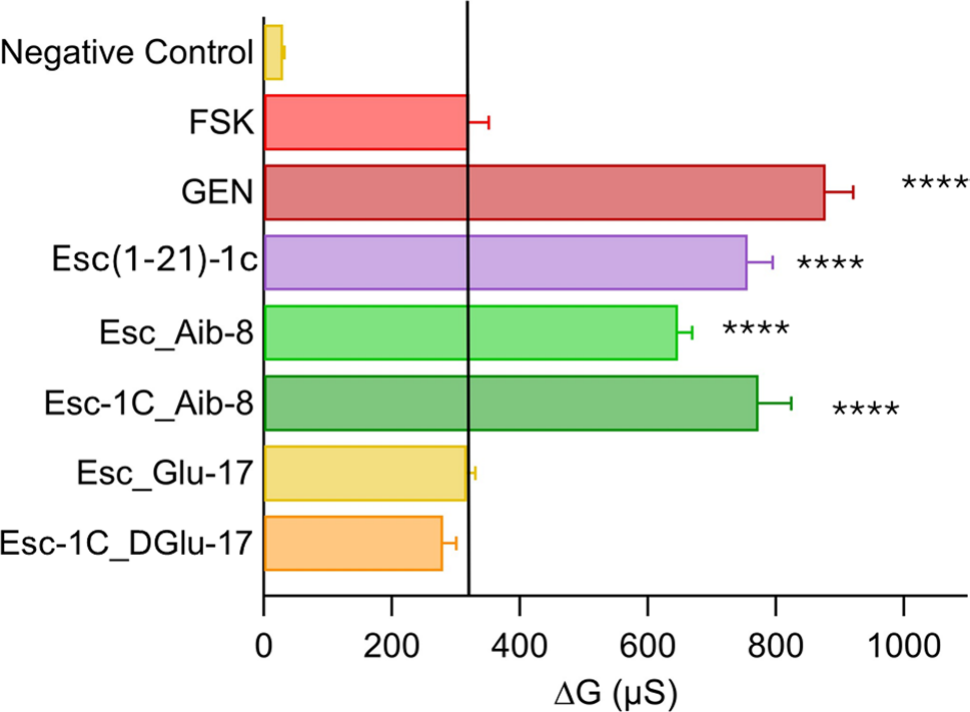
Potentiator effect of peptides on the activity of F508del-CFTR. F508del-FRT epithelium was incubated for 24 h with 1 µM VX-809 (to allow the mutated protein to reach the apical membrane). CFTR-mediated transepithelial conductance was evaluated as the difference between the conductance measured after 10 min addition of 20 µM FSK alone or after addition of the combination FSK+10 µM GEN or + peptides (at 10 µM), and the conductance measured after CFTR-inhibition (delta conductance, ∆G), due to the addition of the specific CFTR inhibitor PPQ102 (30 µM). Cells pre-incubated with VX-809 but not activated were used as negative control. All data are expressed as mean ± SEM from n ≥ 6 independent experiments. The level of statistical significance of samples versus FSK (highlighted by the solid black line) is indicated as follows: **** p < 0.0001

CFTR activity was expressed as delta conductance (∆G). This parameter was calculated as the difference between the transepithelial conductance (TEEC) measured 10 min after stimulation of the epithelium with FSK alone, or with the combination FSK plus GEN/peptides, and the conductance measured after CFTR inhibition. As shown in Fig. 2, compared to FSK-treated samples, no variation of ∆G was recorded for the Glu containing peptides. In contrast, 2-fold higher CFTR-mediated conductance was recorded when the epithelium was exposed to the Aib-carrying analogs, at the concentration of 10 μM. Esc(1–21)−1c was included for comparison.

### Peptides’ effect in homozygous F508del and heterozygous F508del/G542X primary bronchial epithelial cells

**Fig. 3:**
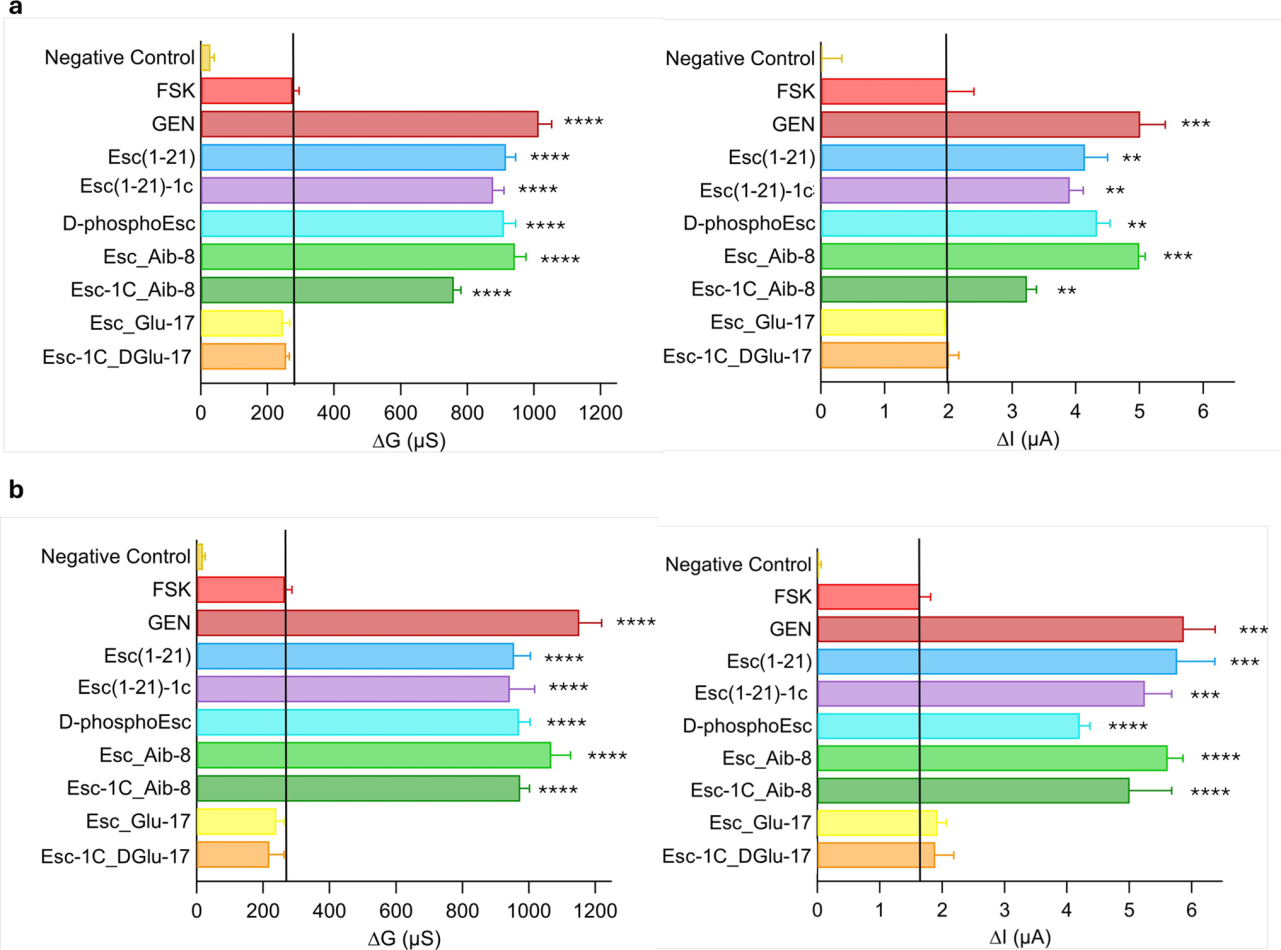
Potentiator effect of AMPs on CFTR in homozygous F508del (**a**) or heterozygous F508del/G542X (**b**) human bronchial epithelial cells. The bar graphs report ∆G and ∆I, calculated as the difference between the conductance (G) or the short-circuit current equivalent (Ieq) measured after 10 min addition of 20 µM FSK alone or after addition of the combination FSK + 10 µM GEN/peptides and the G or Ieq values measured after administration of PPQ102. Cells pre-incubated with VX-809 but not activated were used as negative control. All data are expressed as mean ± SEM from n ≥ 4 independent experiments. The level of statistical significance of samples versus FSK (highlighted by the solid black line) is indicated as follows: ** p < 0.01; *** p < 0.001; **** p < 0.0001

Despite FRT cell lines are largely used for studying the effect of therapeutics on CFTR [10, 37], bronchial epithelial cells represent a more suitable in vitro model to reflect CF lung environment [33]. Therefore, the peptides were analysed on primary homozygous F508del and heterozygous F508del/G542X. A protocol ad hoc for patient collected primary cells was applied [38]. In brief, after addition of each compound (at 10 μM) to the corrected epithelium, both TEEC and transepithelial electrical potential difference (PD) were measured. All values were taken at resting condition; after addition of apical amiloride (to inhibit epithelial sodium channel, ENaC [36]); after maximal stimulation of mutated CFTR with FSK plus GEN/peptide; and after CFTR inhibition (Fig. 3).

From TEEC and PD, the equivalent short-circuit current (*I*eq) was calculated. Epithelia pretreated with VX-809 and not activated were included as control samples. Except for the Glu-containing peptides, which did not show any activity, all the other Esc peptide isoforms were able to significantly increase the activity of the mutated CFTR, like GEN, in both homozygous and heterozygous primary bronchial epithelial cells (Fig. 3 a and b), with an effect which was almost 2–3-fold higher than that provoked by FSK alone. This confirmed the CFTR-potentiator effect of the peptides on primary bronchial epithelial cells in a manner comparable to that found in immortalized FRT cell line.

### Peptides’ effect on the ion current of FRT cells expressing CFTR with different gating mutations

**Fig. 4:**
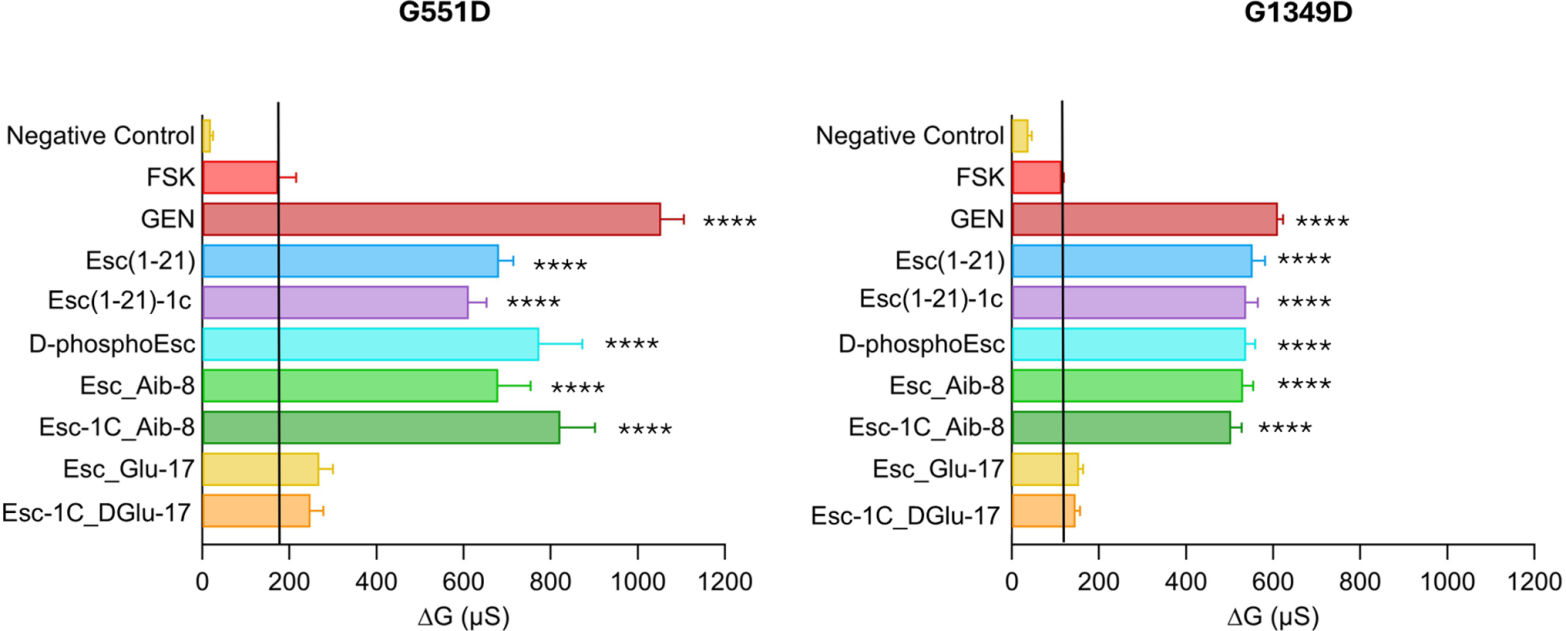
Potentiator effect of peptides on the activity of G551D-CFTR and G1349D-CFTR expressing FRT cells**.** CFTR-mediated transepithelial conductance was evaluated as the difference between the conductance measured 10 min after stimulation with 20 µM FSK, FSK + 10 µM GEN, or FSK + 10 µM peptides, and the conductance measured after CFTR-inhibition (∆G). Untreated cells were used as negative control. All data are expressed as mean ± SEM from n ≥ 6 independent experiments. The level of statistical significance of samples versus FSK (highlighted by the solid black line) is indicated as follows: **** p < 0.0001

Subsequently, to explore whether the potentiator effect of Esc peptides and their analogs was maintained towards CFTR carrying gating mutations belonging to class III, TEER experiments were performed on FRT cells expressing G551D-CFTR and G1349D-CFTR (Fig. 4). Notably, also in this case, no variation in CFTR-mediated conductance was observed for the peptides bearing Glu (Esc_Glu-17 and Esc-1C_DGlu-17). In comparison, the Aib-containing analogs as well as the Esc peptides and the d-phosphoEsc isoform had a similar effect in ameliorating the activity of both CFTR mutants; this effect was at least 3-fold-higher than that of samples treated with FSK alone. These results demonstrate the efficacy of the peptides on CFTR variants that severely affect the mechanism of channel gating.

## Mechanistic studies

### Patch clamp

**Fig. 5:**
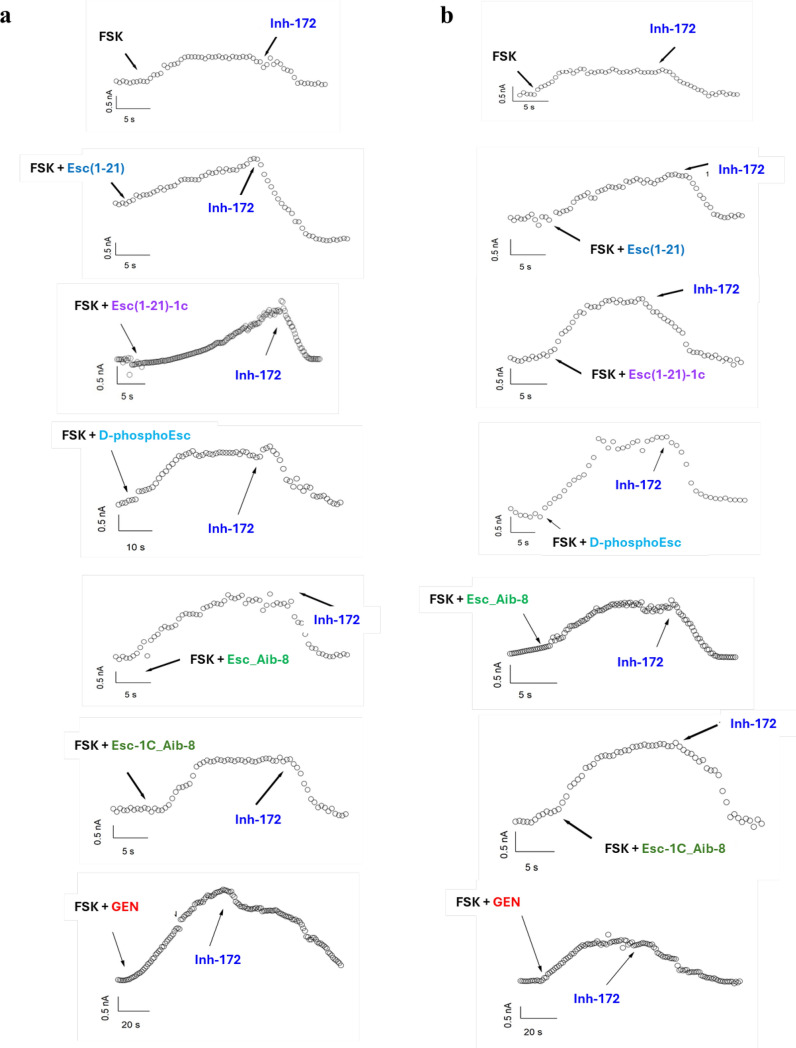
Representative whole-cell membrane time course in G551D (**a**) and G1349D-FRT (**b**) cells. Representative time course of currents elicited in cells during the addition of 20 µM FSK+10 µM peptides or GEN, as indicated, and after subsequent application of 10 µM CFTRInh-172 at + 100 mV

**Fig. 6:**
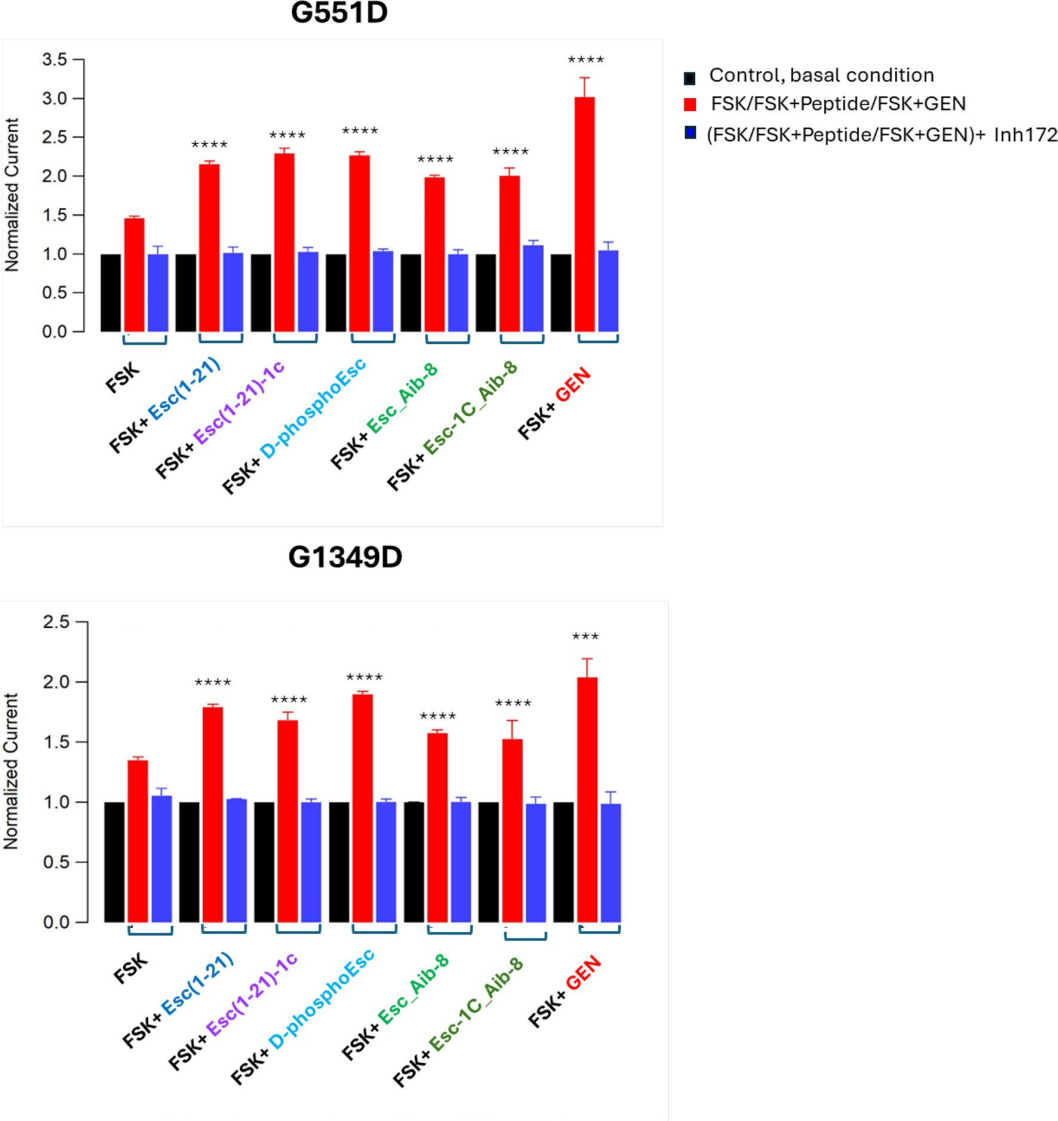
Normalized current from whole-cell membrane experiments in G551D and G1349D-FRT cells. Graph shows the normalized current density measured at +100 mV for indicated treatment (control basal condition, black bars; after CFTR activation, red bars and after CFTR inhibition, blue bars). Data are expressed as mean ± SEM from n ≥ 6 independent experiments. The level of statistical significance of activated samples (red bar) versus FSK is indicated as follows: *** p < 0.001; **** p < 0.0001

The potentiator effect of Esc peptides, d-phosphoEsc and Aib-carrying isoforms was then validated by patch-clamp experiments in whole-cell recordings on FRT cells expressing G551D (Fig. 5 a) or G1349D-CFTR (Fig. 5b) to investigate the electrophysiological response of the mutated CFTR to the selected peptides. The mutated CFTR was activated by FSK in combination with the peptides or GEN that were added to the extracellular solution at a concentration of 10 µM. Perfusion of each peptide elicited large outward currents, with at least 2-fold increase, compared to control basal condition, at the chosen potential of +100 mV at which maximal current intensity was achieved (Fig. 5). Note also that, as for the TEER experiments, the administration of the peptides to the extracellular solution induced a considerable increase of the current intensity (Fig. 6, red bars), compared to that provoked by FSK alone (p < 0.0001), pointing out a substantial recovery of the mutated channel activity, similarly to the effect triggered by the positive control GEN (Figs. 5 and 6). After CFTR inhibition (Fig. 5 and Fig. 6 blue bars), currents reverted almost to the level preceding peptides’ addition (Fig. 5 and Fig. 6, black bars), proving that the current rise caused by the peptides in combination with FSK is due to activation of CFTR. Representative images of superimposed currents triggered at membrane potentials ranging from –100 mV to +100 mV in the external solution before addition of compounds, as well as upon administration of FSK plus peptide/GEN and subsequent addition of the specific CFTR inhibitor 172 are reported in Fig. S1.

### Effect of peptides on intracellular calcium ions

**Fig. 7:**
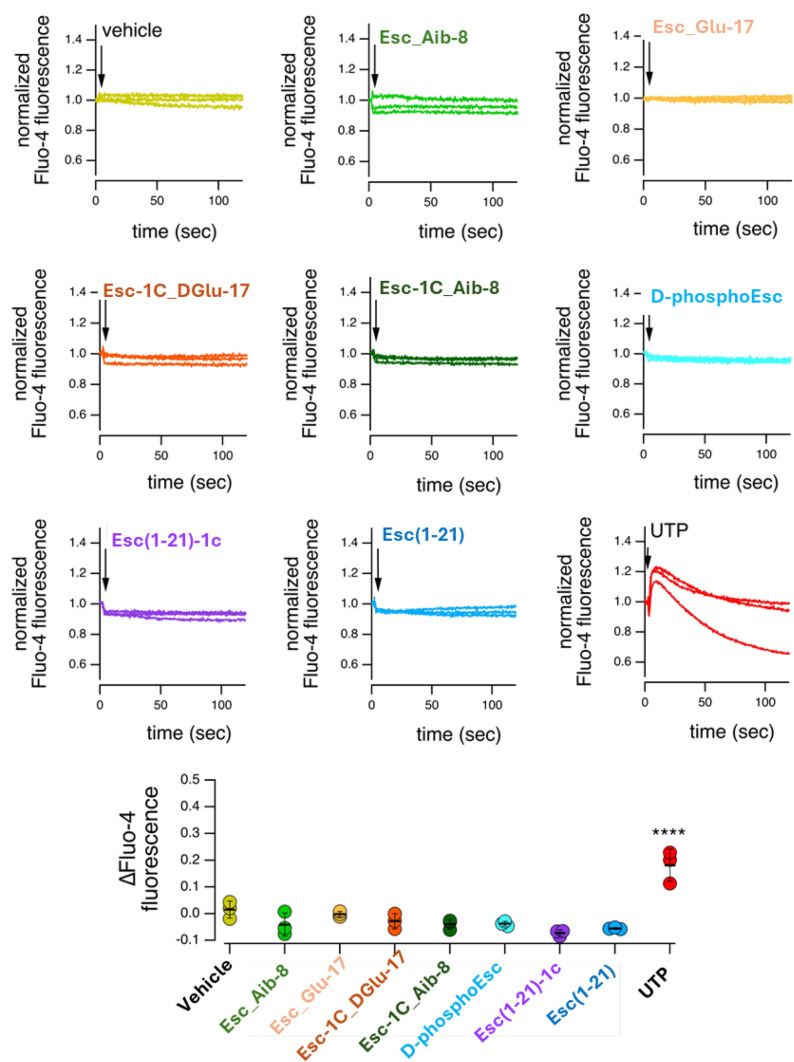
Effect of peptides on intracellular calcium. Representative traces (top) and summary of results (bottom) from experiments with the calcium-sensitive fluorescent probe Fluo-4. Every trace in the graphs represents a single replicate for each condition and reflects calcium variation during time. Traces show the time course of normalized fluorescence (to the background) following addition (arrows) of vehicle, peptides (10 µM each), or UTP (100 µM). The scatter dot plot shows the maximal fluorescence change for each stimulus. **** p < 0.0001 *versus* vehicle

To exclude that the increased peptide-induced ion current could be due to the contribution of other cellular processes, mediated by an increased intracellular concentration of calcium ions, by either a release from intracellular stores or by influx through the plasma membrane, with consequent activation of other ionic channels (i.e. calcium-dependent TMEM16A chloride channel or BK potassium channel) [39], the effect of peptides on intracellular calcium ions was explored. To address this issue, null FRT cells were loaded with the calcium-sensitive fluorescent probe Fluo-4; subsequently, Esc peptides, d-phosphoEsc, Aib-carrying isoforms as well as the inactive Glu-containing peptides were added acutely. UTP (positive control) and vehicle were also included for comparison. Changes in Fluo-4 fluorescence, reflecting changes in intracellular calcium, were monitored with a microplate reader which was also equipped with syringe pumps to add stimuli. As reported in Fig. 7, the effect of all peptides was negligible and essentially identical to that of the vehicle, thus excluding their plausible activation of calcium-dependent chloride channels [39]. In contrast, addition of UTP promoted a marked and rapid increase in fluorescence due to activation of the purinergic signaling cascade and consequent release of calcium from intracellular stores. The effect of FSK on intracellular calcium ions was also evaluated by the Fluo-4 assay in the same experimental conditions used for peptides. Briefly, after cells incubation with Fluo-4, we injected acutely FSK alone or FSK plus each peptide. Dimethyl sulfoxide (DMSO) and UTP were also tested as negative and positive control, respectively. As shown in Fig. S2, any significant change in intracellular calcium mobilization was detected in the presence of FSK, alone or in combination with peptides, confirming that these peptides are not acting on calcium-dependent chloride channels.

### Molecular dynamics (MD) simulations

**Fig. 8:**
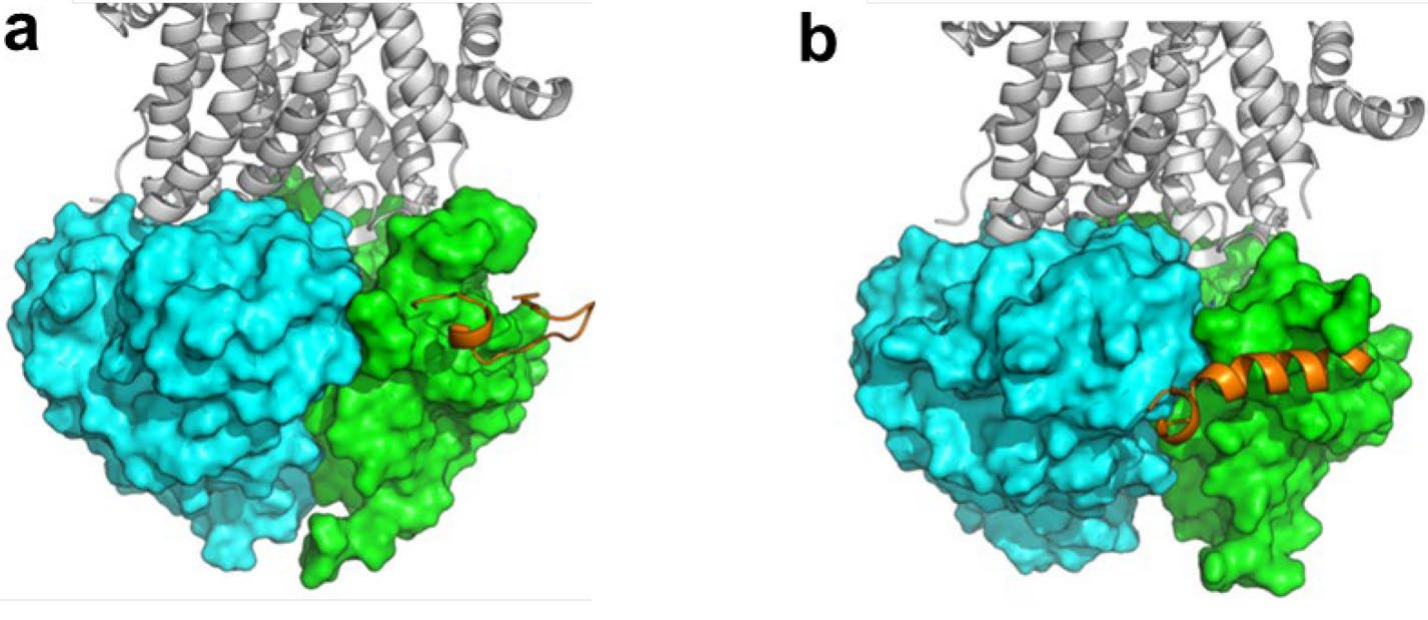
Representative frame extracted from MD simulations by cluster analysis, showing the interaction between Esc_Aib-8 (**a**) and Esc_Glu-17 (**b**) analogs and F508del NBD1/NBD2 heterodimer. The CFTR transmembrane domain has been structurally added on the top of the NBDs heterodimer for the sake of clarity, and it is shown as white cartoon. NBD1 is shown as a green surface, NBD2 is shown as a cyan surface. Peptides are in orange

The possible interaction between the NBD1/NBD2 heterodimer of F508del- and G551D-CFTR and the newly designed analogues of Esc peptides was investigated by molecular dynamics (MD) simulations, according to the procedure described previously [33]. To avoid any conformational or positional bias, the recognition and binding between NBDs and peptides were simulated by placing each peptide and the NBDs heterodimer in a random reciprocal orientation of the simulation box at a non-binding distance (e.g. higher than 40 Å). Then, unbiased MD trajectories were produced for 500 ns for each peptide/CFTR complex. Computational results were confirmed by multiple replicas of MD simulations for each peptide, and they strengthen the initial hypothesis that the NBD1/NBD2 heterodimer is the potential target of AMPs [33]. The binding site was prevalently located on the NBD1, in proximity of the NBD1/NBD2 binding interface, aspreviously observed for Esc(1-21) [33]. Through clustering of MD frames, the most representative frame of the cluster with the highest frames population was extracted from MD trajectories, and it was used in structural discussions. Representative frames of the newly de-novo designed all-L Esc_Aib-8 and Esc_Glu-17 peptides are shown in Fig. 8.

**Fig 9:**
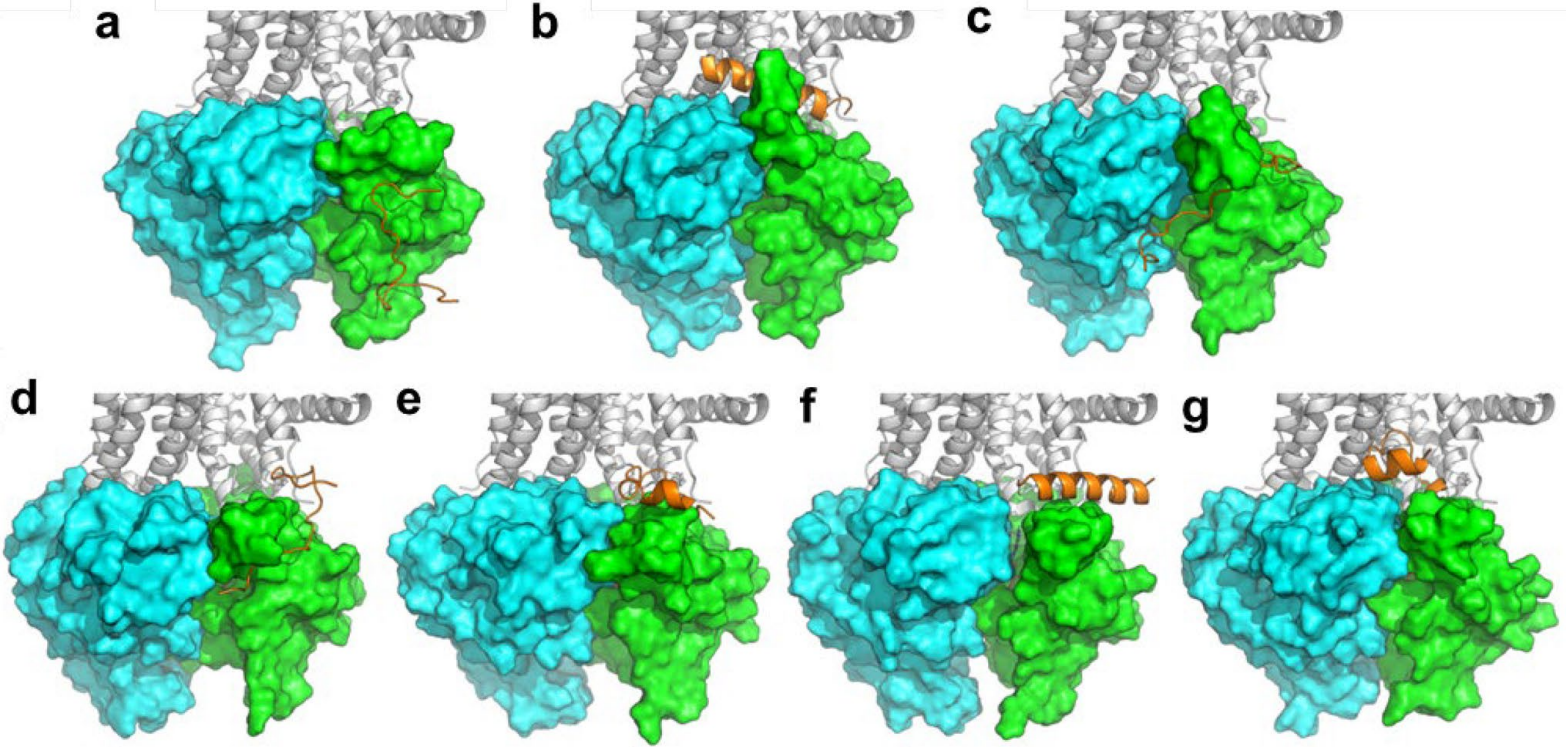
Representative frame extracted from MD simulations by cluster analysis, showing the interaction between the selected peptides and G551D NBD1/NBD2 heterodimer. **a** Esc(1–21); **b** Esc(1–21)−1c; **c** d-phosphoEsc; **d** Esc_Aib-8; **e** Esc-1C_Aib-8; **f** Esc_Glu-17; **g** Esc-1C_DGlu-17. The CFTR transmembrane domain has been structurally added on the top of the NBDs heterodimer for the sake of clarity, and it is shown as white cartoon. NBD1 is shown as a green surface, NBD2 is shown as a cyan surface. Esc peptides are in orange

The analysis of peptides binding to G551D-NBDs, not studied earlier for any peptide, provided similar outcomes such as previously discussed [33]. In fact, all peptides herein investigated by MD simulations bind within the lateral site, in close contact with NBD1 and near its binding interface to NBD2, as depicted in Fig. 9.

**Fig. 10:**
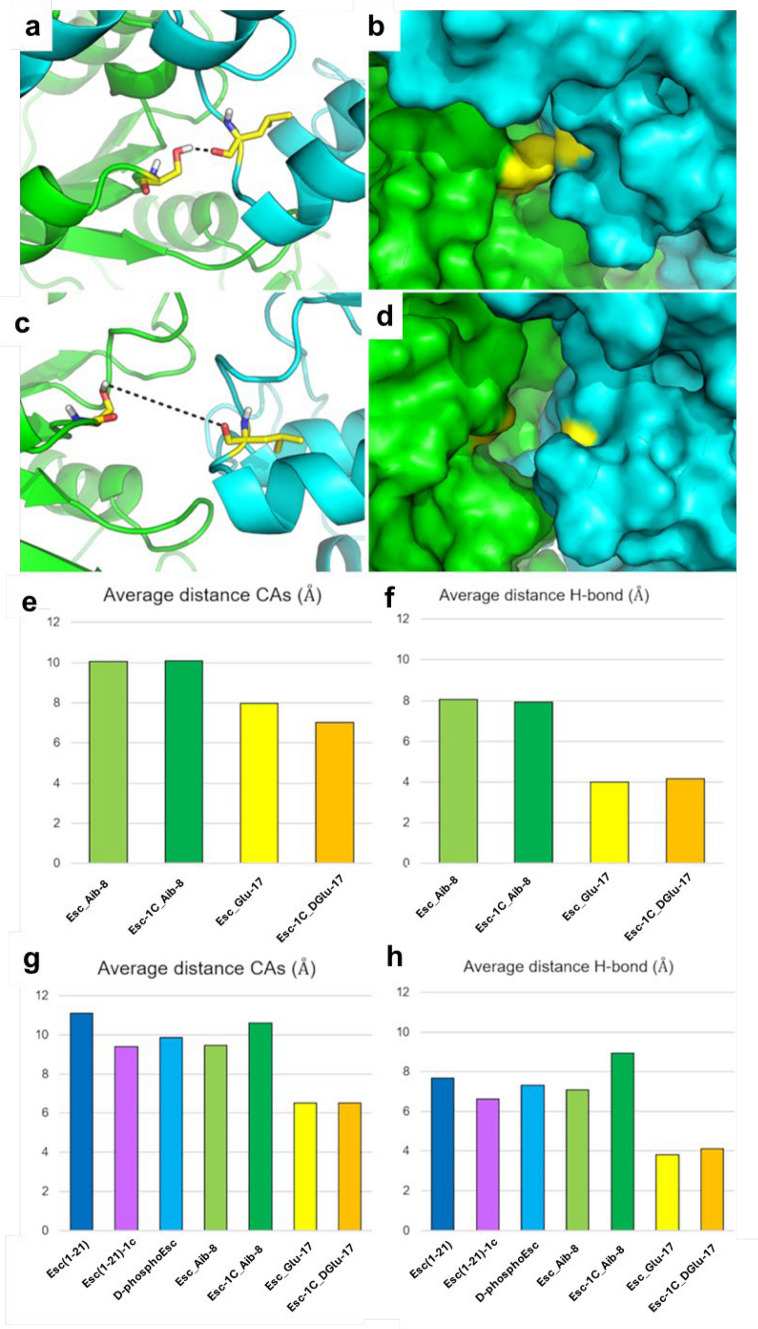
Variation of the distance between Ser605 and Leu1367 at the cytoplasmic end of the CFTR pore. **a-b** representative conformation as identified by MD simulations, showing the intermolecular H-bond between Ser605 and Leu1367 (**a**) and its impact in the separation between the residues at the NBD1/NBD2 cytosolic interface (**b**). **c-d** representative open conformation as identified by MD simulations, showing the non-bonding distance between Ser605 and Leu1367.The two key residues are shown as yellow sticks or yellow surface. NBD1 is coloured in green, NBD2 is coloured in cyan**. e-g** plot of the distances calculated along MD trajectories for Cα and H-bonding atoms of Ser605 and Leu1367 in the F508del NBDs heterodimer (**e-f**) and in the G551D NBDs heterodimer (**g-h**). The same color-codes for peptides as adopted in Figures 2-4 is used

Despite informing on the direct interaction between Esc peptide isoforms and the heterodimeric NBDs of CFTR, binding hypotheses illustrated in Fig. 8 and in Fig. 9 fail to provide a quantitative explanation for the differential potentiator efficacy of the peptides, as observed in vitro. Therefore, to provide additional support to bioactivity results, further investigations were carried out. By visual inspection of MD trajectories, we identified a small region at the cytoplasmic side of NBD1/NBD2 interface that is most variable among different studied systems. Specifically, the structural variation observed in correspondence of the interface between Ser605 (NBD1) and Leu1367 (NBD2) when different peptides were bound to the NBD1/NBD2 heterodimer led us to monitor the distance between these residues along MD simulations. We found that the Ser605-Leu1367 distance nicely correlates with the biological activity data (Fig. 10). In fact, in both mutant CFTR forms studied herein (i.e., F508del, and G551D) the average Cα distance between Ser605 and Leu1367 is around 10 Å (range 9.39–11.11 Å) along MD trajectories upon interaction with peptides showing a remarkable potentiator effect, whereas it decreases to around 7.5 Å (range 6.5–7.97 Å) for the inactive peptides, such as Esc_Glu-17 and Esc-1C_DGlu-17 (Fig. 10 e, g). Similarly, by monitoring the average distance between the side chain of Ser605 (OH group) and the carbonyl backbone of Leu1367, the observed Ser605-Leu1367 distance suggests that these residues are likely H-bonded in the NBDs heterodimer complexed with the ineffective Esc_Glu-17 and Esc-1C_DGlu-17 peptides (Fig. 10 a-b, 10 f, and h).

Finally, calculation of the delta energy of binding of the peptides and NBDs along MD trajectories was conducted by the Molecular Mechanics Generalized Born Surface Area (MM-GBSA) approach. Results further confirm the relative higher affinity of peptides with a potentiator effect compared to peptides endowed with a limited or no activity on the channel, such as Esc_Glu-17 and Esc-1C_DGlu-17 (Table 2), thus reinforcing the hypothesis that the mechanism of action of Esc peptides is based on a direct interaction with the NDBs heterodimer of CFTR.

**Table 2.** Delta energy of binding of Esc peptides to F508del and G551D NBDs heterodimer as calculated along MD trajectories by the MM-GBSA approach, and list of residues within 4 Å from each peptide in the representative MD frame

Peptide	Delta energy of binding against F508del-NBDs (kcal/mol)	F508del NBD1/NBD2 CFTR residues within 4 Å from the peptide	Delta energy of binding against G551D-NBDs (kcal/mol)	G551D NBD1/NBD2 CFTR residues within 4 Å from the peptide
Esc(1–21)	N.D.	N.D.	−34.24 ± 1.79	Thr398, Phe400, Phe405, Phe409, Ser434, Leu435, Leu436, Gly437, Thr438, Pro439, Lys442, Glu479, Ser624, Tyr625, Phe626, Tyr627, Leu636, Gln637.
Esc(1–21)−1c	N.D.	N.D.	−32.27 ± 1.12	Phe400, Trp401, Glu402, Glu403, Gly404, Gly406, Glu407, Leu408, Leu475, Ser1297, Asp1336, Phe1337, Val1338, Val1340, Asp1341, Cys1344.
d-phosphoEsc	N.D.	N.D.	−37.74 ± 1.91	Phe400, Glu402, Glu403, Gly404, Phe405, Gly406, Glu407, Leu408, Phe409, Glu410, Ser434, Leu435, Leu436, Thr438, Pro439, Pro477, Ser478, Glu439, Trp1316, Arg1325, Ser1326, Glu1329, Gln1330.
Esc_Aib-8	−29.56 ± 0.98	Asn396, Thr398, Phe400, Glu402, Phe405, Leu435, Leu436, Gly437, Thr438, Pro439, Lys442, Asp443, Ser478, Glu479.	−30.38 ± 1.66	Phe400, Trp401, Glu402, Glu403, Gly404, Gly406, Glu407, Phe409, Glu410, Ser434, Leu435, Gly437, Thr438, Pro439, Lys442, Glu476, Ser478, Glu479, Glu621, Gln1330.
Esc-1C_Aib-8	−35.24 ± 1.41	Ala457, Gly458, Ser459, Ser605, Lys606, Met607, Leu610, Ile618, His620, Tyr625, Phe630, Leu633, Gln634, Leu636, Gln637, Asp1377, Pro1378, Val1399, Val1447, Pro1451.	−33.13 ± 2.09	Phe400, Glu402, Glu403, Gly404, Phe405, Gly406, Glu407, Leu408, Glu476, Pro477, Ser478, Pro1332, Phe1337.
Esc_Glu-17	−20.32 ± 0.76	Thr398, Phe400, Phe405, Gly406, Glu407, Leu408, Phe409, Leu436, Gly437, Thr438, Pro439, Leu442, Asp443, Ser478, Glu479, Arg1325, Glu1329, Gln1330.	−22.13 ± 0.98	Glu402, Glu403, Gly404, Phe405, Gly406, Glu407, Glu476, Pro1332.
Esc-1C_DGlu-17	−19.12 ± 1.02	Thr398, Ala399, Phe400, Trp401, Glu402, Glu403, Gly404, Phe405, Gly406, Glu407, Leu408, Phe409, Leu435, Leu475, Glu476, Pro477, Ser478.	−16.87 ± 1.13	Trp401, Glu402, Glu403, Phe405, Leu436, Glu473, Pro1332, Gly1333, Asp1336, Phe1337, Val1338, Val1340, Asp1341, Cys1344, Val1345.

*N.D* not determined (peptide interaction not simulated in this work)

In Table 2, residues that are at a potentially binding distance (i.e., < 4 Å from each peptide) in the two CFTR mutants, such as extracted from the most representative MD frame, are also reported.

### Antibacterial and cytotoxic activities

All designed analogs of Esc peptides showing a CFTR potentiator effect were also analyzed for their antibacterial activity by determining the corresponding minimal inhibitory concentration (MIC) towards Gram-negative bacterial strains and the Gram-positive bacterium *Staphylococcus aureus* (which is another relevant bacterial pathogen in CF [40])*,* in comparison to the Glu-containing analogs. Esc(1–21)−1c was included as reference. As indicated in Table 3, the ability to inhibit bacterial growth was significantly reduced in the Esc-1C_DGlu-17 peptide (with a ~4-fold higher MIC) and even more in the phosphorylated peptide, which was almost inactive. In comparison, the Esc_Glu-17 peptide (composed entirely of L-amino acids) exhibited antibacterial activity comparable to the reference Esc(1–21)−1c. These findings suggest that the presence of a negatively charged residue alone is not a critical factor in diminishing the peptide's effectiveness, which likely depends also on its conformational and structural properties. In contrast, the Esc_Aib-8 revealed to be the most active one against Gram-negative bacteria, as pointed out by its lowest MICs, and it was the solely peptide showing activity against Gram-positive bacteria, including *S. aureus,* as recently reported [34].

**Table 3.** Antimicrobial activity of selected peptides

	Esc (1–21)−1c	Esc _Aib-8	Esc1C _Aib-8	d-phosphoEsc	Esc_Glu-17	Esc-1C_DGlu-17
Gram-negatives						
* E. coli* ATCC 25922	12.5	0.78*	3.12	>100	0.78	50
* A. baumannii* ATCC 19606	3.12	0.78*	NT	25	0.78	6.25
* P. aeruginosa* ATCC 27853	6.25	3.12*	6.25	>100	6.25	25
* P. aeruginosa* PAO1	3.12	0.78	3.12	NT	1.56	25
Gram positives						
* S. aureus* ATCC 25923	>100	12.5*	>100	>100	>100	>100
* S. aureus* MDR #1	>100	12.5*	>100	>100	>100	>100
* S. aureus* MDR #2	>100	12.5*	>100	>100	>100	>100
* S aureus* MDR #3	>100	12.5*	>100	>100	>100	>100
* S. aureus* MDR #4	>100	6.25*	>100	>100	>100	>100

MICs are the values obtained from three identical readings of four independent experiments

*NT* not tested

^*^values taken from [34]

**Fig. 11:**
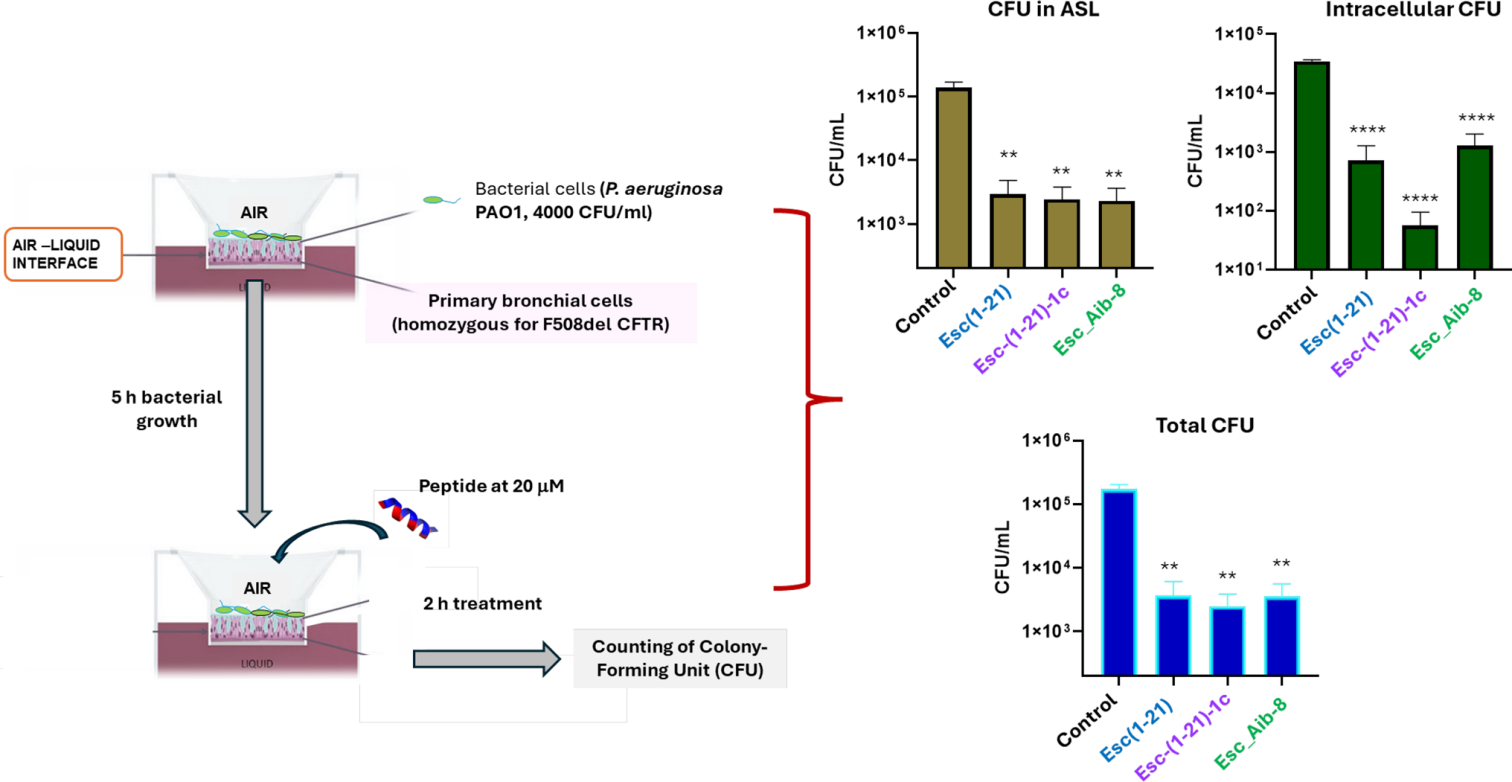
Effect of peptides on the number of *P. aeruginosa* cells grown on the surface of polarized human airway epithelial cells (homozygous for F508del mutation), for six hours (1 h for adhesion plus 5 h for bacterial growth). Two hours after peptide treatment, the number of bacterial cells present in the ASL and intracellularly was enumerated as colony forming units (CFU). The vehicle PBS was used as control. The level of statistical significance of samples *versus* control is indicated as follows: ** p < 0.01; ***p < 0.001; ns, not significant

Due to the importance of bacterial biofilm in resistance to antibacterial treatment by traditional antibiotics, the antibiofilm properties of Esc_Aib-8 in comparison to Esc peptides was examined by the well-established biotic model [41]. This model allowed to evaluate the efficacy of the peptides on bacteria growing on the surfaces of differentiated airway epithelial cells, and likely forming a biofilm community [42, 43]. *P. aeruginosa* PAO1 was used as a reference, being the principal bacterial pathogen responsible for progressive lung disease, especially in patients with CF. The bacterium was allowed to attach to the apical side of polarized airway epithelial cells derived from a homozygous F508del patient and to grow at air-liquid interface (ALI). Afterwards, the coculture was treated with phosphate buffered saline, PBS (control), Esc_Aib-8 or Esc peptides, and the number of bacterial cells was subsequently counted. Interestingly, cocultures treated either with Esc_Aib-8 or Esc peptides had a substantially lower level of *P. aeruginosa* cells (Fig. 11), with approximately a 2-log_10_-unit reduction of bacterial load (including bacterial cells present in the airway surface liquid, ASL, or localized intracellularly), at the modest concentration of 20 μM which did not provoke any significant cytotoxic effect when tested on human bronchial epithelial cells expressing F508del-CFTR (more than 90% viable cells), thus increasing our interest for the development of these peptides as new multifunctional drugs.

### In vivo studies: antipseudomonal effect of peptides in the lung of β-ENaC transgenic mice

**Fig. 12:**
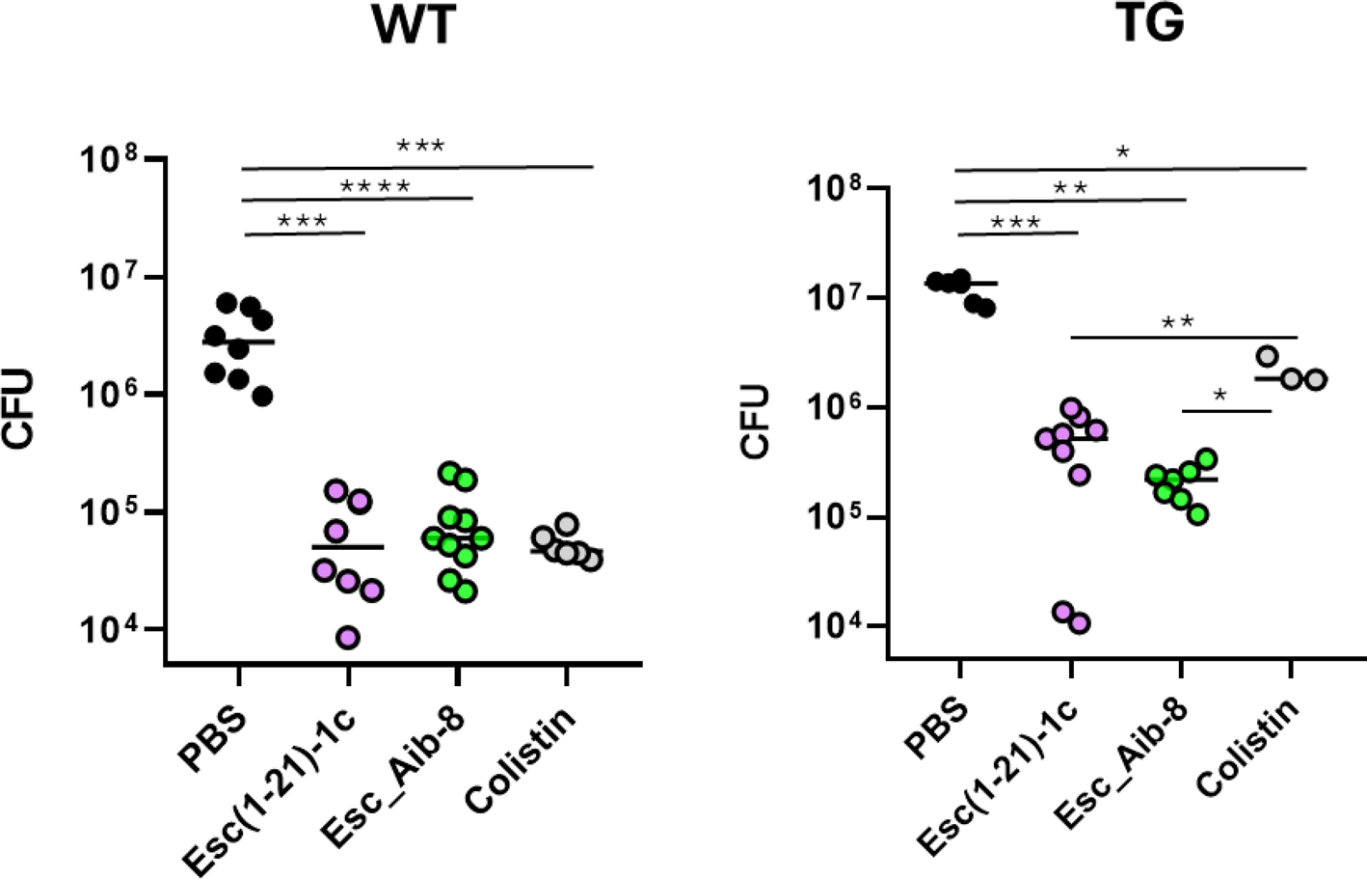
Effect of peptides and colistin on the number of viable *P. aeruginosa* cells (CFU) in the lungs of pulmonary infected β-ENaC mice (TG) and the littermate controls (WT) at 24 h after peptide administration at 0.1 mg/kg (2 μg). Animals were intratracheally infected with 3 x 10^6^ cells. Results are mean ± SEM from three independent experiments. Statistical analysis between different groups is indicted as follows: *p < 0.05, **p < 0.01, *** p < 0.001, **** p < 0.0001

Considering the antibacterial outcome of the selected peptides in the coculture model and the higher in vivo antipseudomonal activity previously shown for Esc(1–21)−1c compared to Esc (1–21) [30, 31], we sought to examine the efficacy of the most promising candidates i.e. Esc_Aib-8 and Esc(1–21)−1c, in reducing lung bacterial burden in proper β-ENaC transgenic mice, following pulmonary infection with *P. aeruginosa.* These animals have an airway-specific overexpression of the ENaC and exhibit CF-like pathological features simulating CF ion transport and airway mucus obstruction [44, 45], compared to wild-type mice with the same genetic background. The clinically used lipopeptide colistin was included for comparison. As illustrated in Fig. 12, in wild-type littermate mice, the Esc_Aib-8 showed similar efficacy to that of Esc(1–21)−1c and colistin, causing ~ 2-log_10_ reduction in the number of lung bacterial cells, 24 h after i.t. administration at a concentration as low as 0.1 mg/kg (corresponding to 20 μM). Interestingly, the activity of the peptides was preserved in transgenic β-ENaC mice, with the Esc_Aib-8 peptide having a trend slightly stronger than that of Esc(1–21)−1c. Note also that, according to what reported in [45], *P. aeruginosa* burden in the lungs of untreated transgenic β-ENAC mice was significantly more pronounced than in wild-type littermates (WT, Fig. 12), likely due to the slower bacterial clearance of β-ENaC mice, thus making the peptide’s action even harder. Most importantly, the selected peptides resulted to be significantly more efficient than colistin in lowering bacterial load in lungs mimicking CF-like airway mucus obstruction.

## Discussion

CF is a multi-organ illness, but failure of the respiratory system represents the principal problem. Indeed, the dysregulated transport of ions through the airway’s epithelia negatively affects the mucociliary clearance, entrapping inhaled microorganisms, with the establishment of a persistent pulmonary infection and the development of a hyperinflammation that remains the major cause of morbidity and mortality [46–48]. Despite traditional therapies, based on small organic molecules, aim at promoting the correct folding of CFTR or enhancing ions transport [49–52], prevention and treatment of lung infections is still severely challenging. Remarkably, nowadays AMPs are described as a promising new class of anti-infective agents with expanding properties [53]. We recently discovered a novel feature of frog-skin derived AMPs, that is the capability to ameliorate the function of F508del-CFTR, with an efficacy comparable to that of the known potentiator GEN. However, nothing has been reported i) on the effectiveness of these peptides on other CFTR variants, including those that severely affect the mechanism of channel opening (like G551D), along with their plausible mode of action, or ii) their antimicrobial activity in conditions mimicking CF-lung.

It is generally accepted that opening of CFTR channel occurs upon phosphorylation of its R domain and NBD dimerization induced by ATP binding, while gate closure is promoted by separation of the NBDs, following ATP hydrolysis [7, 8, 54]. Although the exact mechanism coupling NBD dimerization to the movement of the gate in the TMDs is still argued [55], any compound that provides a favourable condition for ATP binding, formation of NBD dimer and opening of the TMD gate could act as a CFTR potentiator. Thanks to cryogenic electron microscopy studies of CFTR, two distinct conformations were identified [56]. Without phosphorylation and ATP, CFTR forms a pore-closed conformation in which the R domain sterically prevents dimerization of the NBDs which remain separated by approximately 20 Å [56]. In comparison, in the phosphorylated and ATP-bound CFTR, the NBDs form a closed dimer with the two ATP molecules bound at the NBDs interface [56]. Liu and colleagues demonstrated that Ivacaftor (VX-770) interacts with CFTR at the protein-lipid interface [57]. This binding occurs within a groove created by the transmembrane helices (TM) 4, 5, and 8; and it coincides with a hinge region within TM8, involved in gating [57]. When ATP binds to CFTR, the extracellular portion of TM 8 undergoes a rotational movement around this hinge. The drug's effectiveness may be attributed to the stabilization of this rotational motion [52].

In our work, the finding of i) a significant increase of TEEC and current intensity in cells expressing G551D/G1349D-CFTR, upon exposure to the peptides in the presence of FSK, followed by a return of the conductance/current amplitude to the value measured before activation of the channel; and ii) the lacking enhancement of intracellular calcium level in cells exposed to the selected peptides, contributed to strength the statement that the increased peptide-induced membrane current is mediated by activation of CFTR rather than other processes related to an increased concentration of calcium ions, including the activation of calcium-dependent chloride channels, like TMEM16A [58].

Furthermore, computational studies have strongly suggested that Esc peptides and selected analogues work as potentiators of CFTR with gating defect, by direct interaction with the cytosolic NBDs, followed by distancing residues located at the cytosolic side of the NBD1/NBD2 interface, Ser605 and Leu1367, regardless of the binding position of the peptide. This would presumably contribute to stabilizing the open conformation of the channel, thus favouring an increased ion current through CFTR. In other words, we observed a new local phenomenon consisting in the turn of Ser605 which does not longer make an H-bond with Leu1367, upon binding of the peptides endowed with a potentiator activity, to the NBDs heterodimer. This could have a functional role by better defining and stabilizing the channel through which the ions pass. Overall, MD study has added further insights into the mechanism of action of Esc peptides, providing atomistic and thermodynamic details of their binding to the NBDs heterodimer in F508del and G551D CFTR mutants. We rationalized that the structural effect of AMPs on mutant NBD1/NBD2 heterodimers might be localized at the cytoplasmic end of the pore, highlighting the Ser605-Leu1367 distance as one of the key determinants to monitor the potentiator effects of the peptides. Indeed, the results of MD analysis correlate with biological activity data, as well as with electron microscopy structural data of the F508del-CFTR in complex with small molecules endowed with potentiator efficacy such as elexacaftor (VX-445) (PDB-ID: 8EIG; Cα distance = 11.06 Å; H-bond distance = 7.32 Å) as well as with the wild-type phosphorylated and ATP-bound CFTR (PDB-ID: 6MSM; Cα distance = 8.61 Å; H-bond distance = 6.54 Å) [59, 60]. Computational studies do also correlate with the results of thermodynamic analysis showing that the peptides displaying a CTFR potentiator effect have a theoretical binding affinity to CFTR higher than that obtained for peptides devoid of such effect.

Notably, a peptide-based approach to restore the activity of CFTR mutants is quite new, in the CF field. Analogs corresponding to fragments of the peptide neurotoxin, named crotoxin, were lately shown to exhibit a potentiator effect on F508del-CFTR, targeting the NBD1 domain [61]. Moreover, a cell permeable phosphoinositide-3 kinase γ mimetic peptide was discovered to safely increase cAMP in the lungs and to promote the phosphorylation of F508del-CFTR, triggering channel gating [62].

However, to the best of our knowledge, except for Esc peptides, no other cases of peptide molecules displaying a potentiator effect on CFTR carrying class III mutations have been described.

In addition, unlike other CFTR modulators active on class II and III mutations, Esc peptides are also able to tackle Pseudomonas lung infections. In this work, by using a biotic biofilm assay in which *P. aeruginosa* was grown on the surface of primary airway epithelial cells expressing F508del-CFTR, a significant reduction of bacterial cells (~2 log_10_ reduction) was obtained 2 h after treatment of the co-culture with Esc peptides or Esc_Aib-8, at 20 μM. Note that studies performed by Chen and co-authors demonstrated the efficacy of the artificial peptide WLBU2 in preventing biofilm formation of *P. aeruginosa*, when applied to the surface of healthy bronchial epithelial cells at a comparable concentration (16 μM), but no activity of killing of *P. aeruginosa* cells grown in contact with CF bronchial epithelial cells was reported [41].

Importantly, although several AMPs have been tested in vitro, in CF lung sputum, or in vivo, in animal models of chronic infections [41], no studies have been carried out to evaluate the antimicrobial/antipseudomonal efficacy of AMPs in models that better reflect the CF lung environment characterized by mucus plugging and chronic neutrophils inflammation. To this purpose, β-ENaC transgenic mice allow to get insight into the pathophysiology and treatment of mucus-obstruction lung disease and represent a suitable model for testing the in vivo efficacy of therapeutics against infections or for evaluating their rate of bacterial clearance in CF-like lung [63]. When β-ENaC transgenic mice were intratracheally infected with *P. aeruginosa* and then treated with the most promising peptides for the dual CFTR potentiator effect and antimicrobial activity (i.e. Esc(1–21)−1c and Esc_Aib-8), these latter were found to retain their antimicrobial efficacy, causing ~2 log_10_ lowering of lung bacterial burden, as in the littermate, when intratracheally administered at 20 μM. Moreover, in β-ENaC transgenic mice, used to simulate CF-lung (in terms of altered ions transport, depletion of airways surface liquid and impaired mucociliary clearance), both peptides displayed a better antimicrobial efficacy than that of the clinically used colistin (Fig. 12).

In summary we have provided the first evidence of the capability of Esc peptides and the analog with anti-staphylococcus activity i.e. Esc_Aib-8, i) to improve the function of CFTR carrying different conductance defects, through a novel molecular mechanism likely based on the stabilization of the open conformation of the pore at its cytosolic end, upon peptides’ binding to the NBDs heterodimer; ii) to preserve their CFTR potentiator effect on primary bronchial epithelial cells, regardless of their homozygous or heterozygous F508del mutation in the CFTR gene. This is a relevant aspect, considering that several CFTR modulators have not been effective in F508del heterozygotes or carrying nonsense mutations [64]; and (iii) to maintain their antibacterial activity (using *P. aeruginosa* as a model microorganism) under conditions mirroring CF lung.

Therefore, these results highly encourage future research in optimizing the development of AMPs as new multifunctional drugs with a large spectrum of antibacterial activity, especially for treatment of CF lung disease, either when used alone or in combination with currently used modulators.

## Materials and methods

### Cells and peptides

FRT cells with no CFTR expression (null FRT) or with a stable expression of mutated CFTR (F508del-FRT, G551D-FRT, G1349D-FRT) as well as bronchial epithelial cells expressing a functional copy of CFTR (wt-CFBE41o-) were used [65]. FRT and CFBE41o- cells lines were maintained at 37 °C in a 5% CO_2_ humidified atmosphere, in appropriate medium supplemented with 2 mM L-glutamine, 10% fetal calf serum, 100 U/ml penicillin, and 100 μg/ml streptomycin: Coon’s modified Ham’s F-12 medium (Sigma-Aldrich, St. Luis, MO) for FRT cells and minimal essential medium (MEM, Euroclone, Italy) for CFBE41o- cells. Primary human bronchial epithelial cells derived from CF individuals were provided by the Italian Cystic Fibrosis Foundation (FFC) Cell Culture Service. They were grown in proliferative serum-free medium containing 1:1 mixture of LHC basal medium and RPMI 1640 (Life Technologies, Monza, Italy) with the addition of hormones, supplements and antibiotics (100 U/ml penicillin and 100 μg/ml streptomycin).

Esc peptides and analogous were purchased from Biomatik (Wilmington, USA). The synthesis was conducted by a stepwise solid-phase technique and F-moc protocol. Purification up to 95% was achieved through reverse-phase high performance liquid chromatography and the molecular mass was confirmed using mass spectrometry. Colistin sulphate was purchased from Sigma (St. Louis, MO).

### Immunofluorescence

About 7 × 10^5^ FRT cells suspended in their standard culture medium were seeded on 0.13- to 0.17-mm-thick coverslips properly placed into 35-mm dish plates and incubated overnight at 37 °C and 5% CO_2_. Afterwards, null FRT and F508del-FRT cells were treated with 3 μM VX-809 in medium supplemented with 10% FBS for 48 h. In parallel, wt-FRT cells and F508del-FRT cells were maintained in the medium without any treatment. Then, cells were fixed with 4% formaldehyde (Sigma-Aldrich, St. Louis, MO, USA) in PBS for 20 min and permeabilized with 0.2% Triton X-100 (Sigma-Aldrich) in PBS for 5 min as in [66]. Cells were stained with anti-CFTR antibody, clone MM13-4 (Sigma-Aldrich), followed by staining with Cy3/peroxidase-conjugated secondary antibody (Jackson Immunoresearch, Philadelphia, PA, USA). Actin was visualized using Alexa-Cy3 conjugated phalloidin (Thermo Fisher, Waltham, MA, USA). Nuclei were stained with 4′,6-diamidino-2-phenylindole (DAPI, Thermo Fisher). Coverslips were mounted in Prolong Gold antifade (Life Technologies, Carlsbad, CA, USA) and images acquired using a confocal microscope (Leica TCS SP2, Wetzlar, Germany) at 40x objective. Quantification of CFTR signal was performed using ZenBlue Software 3.0 by Celldiscoverer 7 from Zeiss (Oberkochen, Germany). A minimum of 4 fields per sample (at least 150 total cells per sample) from 3 independent experiments were analysed.

### TEER/ PD to evaluate CFTR activation

To evaluate the effect of the peptides on the ion current controlled by CFTR, the transepithelial conductance was measured in epithelia formed by FRT expressing CFTR with different mutation. Cells were seeded on permeable supports (24 Millicell plates PSHT01QR1) and then incubated in their standard culture medium. Cells expressing F508del mutation were pretreated with 1 μM VX-809 to deliver the defective protein to the plasma membrane. After 24 h, the medium was replaced with saline solution containing (in mM): 130 NaCl, 2.7 KCl, 1.5 KH_2_PO_4_, 1 CaCl_2_, 0.5 MgCl_2_, 10 glucose, 10 Na-Hepes (pH, 7.4), which was added to both apical and basolateral compartments of the permeable supports [67]. Initially, TEER was measured in basal conditions without CFTR activation, using the epithelial voltmeter (MILLICELL ERS-2, Millipore Burlington, MA). Afterwards, 20 μM FSK + 10 μM GEN (as positive control) or FSK+ peptides at the desired concentration were added on the apical side of the epithelium and TEER measurement was performed at 37 °C after 10 min from the addition of the compounds. Finally, TEER was measured after the addition of 30 μM PPQ102 to the apical side of the epithelium, to inhibit CFTR. From the values of TEER obtained before and after CFTR inhibition, the CFTR-dependent TEER was calculated, for each condition. All TEER values were converted to transepithelial conductance (TEEC) using the formula TEEC = 1/TEER [68]. F508del-FRT monolayer pretreated with the vehicle dimethyl sulfoxide (0.1% v:v, final concentration), used to solubilize VX-809 [67], and then treated with FSK+GEN did not manifest any activity (data not shown).

For primary bronchial cells, the CFTR activity was measured in Coon’s modified Ham’s F12 medium with 20 mM Na-Hepes (pH 7.3). After equilibration, TEER and PD were measured in each well under basal conditions, after ENaC channel inhibition with apical amiloride (10 μM), after CFTR stimulation with FSK (20 μM) on both apical and basolateral sides, after apical addition of GEN or peptides (10 μM), and after CFTR inhibition with apical PPQ102 (30 μM). Each treatment was left for 10 min before recording electrical parameters. Values of TEER and PD were converted into short-circuit current equivalent using Ohm’s law [36].

### Patch-clamp experiments

Whole-cell membrane currents were recorded in FRT cells stably expressing mutated CFTR (F508del, G551D and G1349D variants); F508del-FRT cells were incubated with 1 μM of the corrector VX-809 for 24 h to increase mutated CFTR membrane expression [69]. The extracellular (bath) solution contained (in mM): 150 NaCl, 1 CaCl_2_, 1 MgCl_2_, 10 glucose, 10 mannitol, 10 Na-HEPES (pH 7.4). The pipette (intracellular) solution contained (in mM): 120 CsCl, 10 TEA-Cl, 0.5 EGTA, 1 MgCl_2_, 10 Cs-HEPES, 40 mannitol, 1 ATP. The channel phosphorylation was determined by the addition of 20 μM FSK in the extracellular solution. To test the potentiator activity we added FSK + 10 μM GEN (positive control) or FSK+ 10 μM peptides to the bath solution in patch-clamp experiments. Each cell was subjected to this stimulation protocol: voltage steps of 500 ms from −100 to + 100 mV, starting from a resting potential of −60 mV with subsequent increments of 20 mV.

### Microplate reader-based Ca^2+^ assay

Experiments for the evaluation of intracellular calcium mobilization were performed using an automated micro-plate reader (FluoStar) equipped with syringe pumps and optical excitation/emission filters (500/535 nm). FRT cells were cultured until confluence in 96-well microplates. Before starting the assay, cells were washed 3 times with complete PBS (200 μl/wash) and then loaded for 1 h at 37 °C with 5 μM Fluo-4/AM (Thermo Fisher Scientific) in PBS containing 10 mM glucose, 0.5 mM sulfinpyrazone and 1% fetal bovine serum. After loading, cells were washed twice with PBS (120 μl/wash) and incubated for 15 min at 37 °C with 100 μl of PBS plus 10 mM glucose and 0.5 mM sulfinpyrazone [70]. The assay was run on one well at a time by programming the plate reader to acquire the fluorescence emitted from each well every 0.2 s for 120 s. At 2.8 s from the start of reading, the plate reader injected 100 μl of PBS (with glucose and sulfinpyrazone) containing vehicle alone (DMSO), peptides (10 µM each) or UTP (10 µM).

### Molecular dynamics simulations

Atomistic simulations were conducted by adopting the protocol described previously [33]. Briefly, the interaction between peptides and the NBDs heterodimer was investigated by unbiased and unrestrained MD simulations in which each peptide and the respective mutant NBD1/NBD2 complex were included in a rectangular box of TIP3P type water molecules, at a distance higher than 40 Å. The total charge was neutralized by the addition of ions (e.g., Na^+^, Cl^-^) and each system was first relaxed by two energy minimization runs: i) energy minimization of the solvent, while keeping the solute as frozen, for 500 steps with the steepest descent (SD) algorithm followed by 2500 steps with the conjugate gradient (CG) algorithm; ii) energy minimization of the solvated solute for 1000 steps with the SD and subsequent 5000 steps with the CG. Each system was then heated to 300 K using the Langevin thermostat at constant volume for 1 ns; systems’ density was equilibrated with the Berendsen barostat at constant pressure for 1 ns, before running a preliminary MD simulation at constant pressure for 50 ns. The final MD trajectories production was run at constant pressure for 500 ns, using a time-step of 2 fs. The AMBER ff15ipq-m force field, which includes parameters for protein mimetics such as the Aib [71] was used for NBDs and peptides, while the General Amber Force Field (GAFF) was used for ATP molecules [72]. Force field parameters for d-configured amino acids were manually derived from the corresponding l-amino acids. MD simulations were run with AMBER 20 [73, 74] while analysis of MD trajectories was carried out with the CPPTRAJ software, which was also used to calculate atomic distances [74]. Up to three independent MD replicas of 500 ns were run for each system.

### Antibacterial activity

#### Determination of MIC

Peptides were tested against the Gram-negative *Escherichia coli* ATCC 25922, *Ainetobacter baumannii* ATCC 19606, *P. aeruginosa* ATCC 27853, *P. aeruginosa* PAO1; and the Gram-positive *S. aureus* ATCC 25923, MDR#1, MDR#2, MDR#3 and MDR#4. The MIC values were determined as previously reported [34]. Briefly, bacteria were grown at 37 °C in Luria-Bertani broth (LB) until an optical density (OD) of 0.8 was reached (λ=590 nm). Aliquots of bacteria (50 µL) were added to each well of a 96-multiwell plate containing serial dilutions of the tested peptides in Mueller Hinton broth (MH). The final cell concentration was 1x10^6^ CFU/ml. The plate was incubated at 37 °C overnight and the MIC was defined as the lowest concentration of peptides able to visually inhibit microbial growth (absence of turbidity) after 16–18 h.

#### Co-culture method on airways epithelium

This assay was performed using co-cultures of polarized and well-differentiated human bronchial epithelial cells derived from a homozygous F508del patient and *P. aeruginosa* PAO1 grown to a log phase. Polarized human epithelia in transwell permeable supports and maintained in ALI culture were inoculated with *P. aeruginosa* PAO1 in 50 µl of cell culture medium (initial inoculum of 200 bacterial cells per ALI culture insert, according to [75]) at the apical side of the supports for 1 h to allow attachment of the bacteria to the epithelium in ALI culture. Unattached bacteria were removed and the supports incubated (37 °C; 5% CO₂) for 5 h prior to the addition of peptide. Each peptide, at 20 μM, diluted in 100 µl PBS, was added for 2 h. After a total of 8 h, the airways surface liquid was collected and aliquots poured on plates for counting. Afterwards, the epithelium was disrupted by adding 150µl Tripsin-EDTA and incubated for 15 min. Subsequently, 200 µl Triton X-100 (0.1%) was added and the supports incubated again for 15 min. Serial dilution and counting of bacterial cells was performed on tryptic soy agar plates to determine the number of CFU.

### Cell viability assay

The Cell Counting Kit 8 (CCK-8) assay was performed as follows to assess cell viability. About 1×10^4^ F508del-CFBE41o- cells were seeded in each well of a 96-well plate and incubated at 37 °C and 5% CO_2_. After 24 h, the medium was replaced by MEM supplemented with 2 mM L-glutamine and containing each peptide at different concentrations [76]. The plate was incubated for 24 h and subsequently 10 μl of CCK-8 reagent were added to each well. After 2 h incubation, the absorbance of each well was measured at 450 nm using a microplate reader (Infinite M200; Tecan, Salzburg, Austria).

### Murine infection model

The animal experiments were performed according to a protocol (protocol 23083347) approved by the Institutional Animal Care and Use Committee (IACUC) of the University of Pittsburgh based on the NIH guide for the care and use of laboratory animals. Transgenic mice with expression of the protein-coding region of the mouse Scnn1b (β-ENaC) and driven by rat airway-specific Scgb1a1 (also called the Clara cell secretory protein) gene were previously described and obtained from Dr. Wanda O'Neal from the University of North Carolina at Chapel Hill [44]. β-ENaC mice in C57BL/6 genetic background were maintained in heterozygous conditions and continuously bred with C57BL/6 wild-type mice. Genotyping was performed for all pups to identify their respective genotypes. Mice expressing β-ENaC and their wild-type littermates at the same ages were used for experiments. Seven-week-old female and male mice were anesthetized by isoflurane inhalation and instilled intratracheally with ~3 x 10^6^ CFU of *P. aeruginosa* PAO1 in 50 µl PBS. One hour after exposure, peptide or colistin was intratracheally administered at 0.1 mg/kg (predetermined on previous studies that demonstrated efficacy) in 50 µl of PBS. Control mice were given 50 µl of PBS without peptide. After 24 h, lungs were harvested, four right lobes of each mouse lung were homogenised, serial diluted to determine the total lung bacterial burden, according to the published method [42].

## Statistical evaluation

Quantitative data from independent experiments were reported as the mean ± standard error of the mean (S.E.M.) Statistical significance was calculated using Student’s t test or ANOVA (GraphPad Prism 8). P values < 0.05 were statistically significant. For electrophysiological experiments (TEER /PD measurements and patch-clamp experiments), comparison between mean data was done using Student’s t test and statistical analysis was carried out using IgorPro 8.0.4 software.

## Supplementary Information

Below is the link to the electronic supplementary material.

Supplementary file1 (PDF 773 KB)

## Data Availability

All data are available upon request to the authors.

## Declarations

### Research involving human and/or animal participants

The study on animals was conducted according to a protocol (protocol 23083347) approved by the Institutional Animal Care and Use Committee (IACUC) of the University of Pittsburgh based on the NIH guide for the care and use of laboratory animals.
