# Supplementary material for: Esc peptides and derivatives potentiate the activity of CFTR with gating defects and display antipseudomonal activity in cystic fibrosis-like lung disease
Source: Cell Mol Life Sci. 2025 Mar 18;82(1):121. doi: 10.1007/s00018-025-05633-9 (PMC11920571; doi:10.1007/s00018-025-05633-9)
Supplement: Supplementary file 1 — Supplementary file1 (PDF 773 KB) [file 18_2025_5633_MOESM1_ESM.pdf]

Supplementary Information

Esc peptides and derivatives potentiate the activity of CFTR with gating defects and display antipseudomonal activity in cystic fibrosis-like lung disease

Loretta Ferrera[1], Floriana Cappiello[2], Arianna Venturini[3], Hexin Lu[4], Bruno Casciaro[2], Giacomo Cappella[2], Giulio Bontempi[5], Alessandra Corrente[2], Raffaele Strippoli[6], Federico Zara[1,7], Y. Peter Di[4]*, Luis J.V. Galietta[3,8], Mattia Mori[9]* and Maria Luisa Mangoni[2]*

[1]UOC Genetica Medica, IRCCS Istituto Giannina Gaslini, Genoa, Italy;
[2] Laboratory Affiliated to Pasteur Italia-Fondazione Cenci Bolognetti, Department of Biochemical Sciences, Sapienza University of Rome, Rome, Italy;
[3] Telethon Institute of Genetics and Medicine (TIGEM), Pozzuoli, Naples Italy;
[4] Department of Environmental and Occupational Health, University of Pittsburgh, Pittsburgh, USA;
[5] Department of Molecular Medicine, Sapienza University of Rome, Rome, Italy;
[6] National Institute for Infectious Diseases L. Spallanzani, IRCCS, Via Portuense, 292, 00149 Rome, Italy
[7] Department of Neurosciences, Rehabilitation, Ophthalmology, Genetics, Maternal and Child Health (DiNOGMI), University of Genoa, Genoa, Italy
[8]Department of Translational Medical Sciences, University of Napoli "Federico II", Naples, Italy;
[9]Department of Biotechnology, Chemistry and Pharmacy, University of Siena, Siena, Italy.

* Corresponding authors:
Maria Luisa Mangoni; Email: marialuisa.mangoni@uniroma1.it
Mattia Mori; Email: mattia.mori@unisi.it
Y. Peter Di; peterdi@pitt.edu

This PDF file includes:

Supplementary Text
Fig. S1
Fig. S2

Supplementary Text

Patch-clamp experiments in FRT cells. Whole-cell membrane currents were recorded in FRT cells expressing G551D and G1349D CFTR by using the same experimental solutions and stimulation protocols as described in the main text.

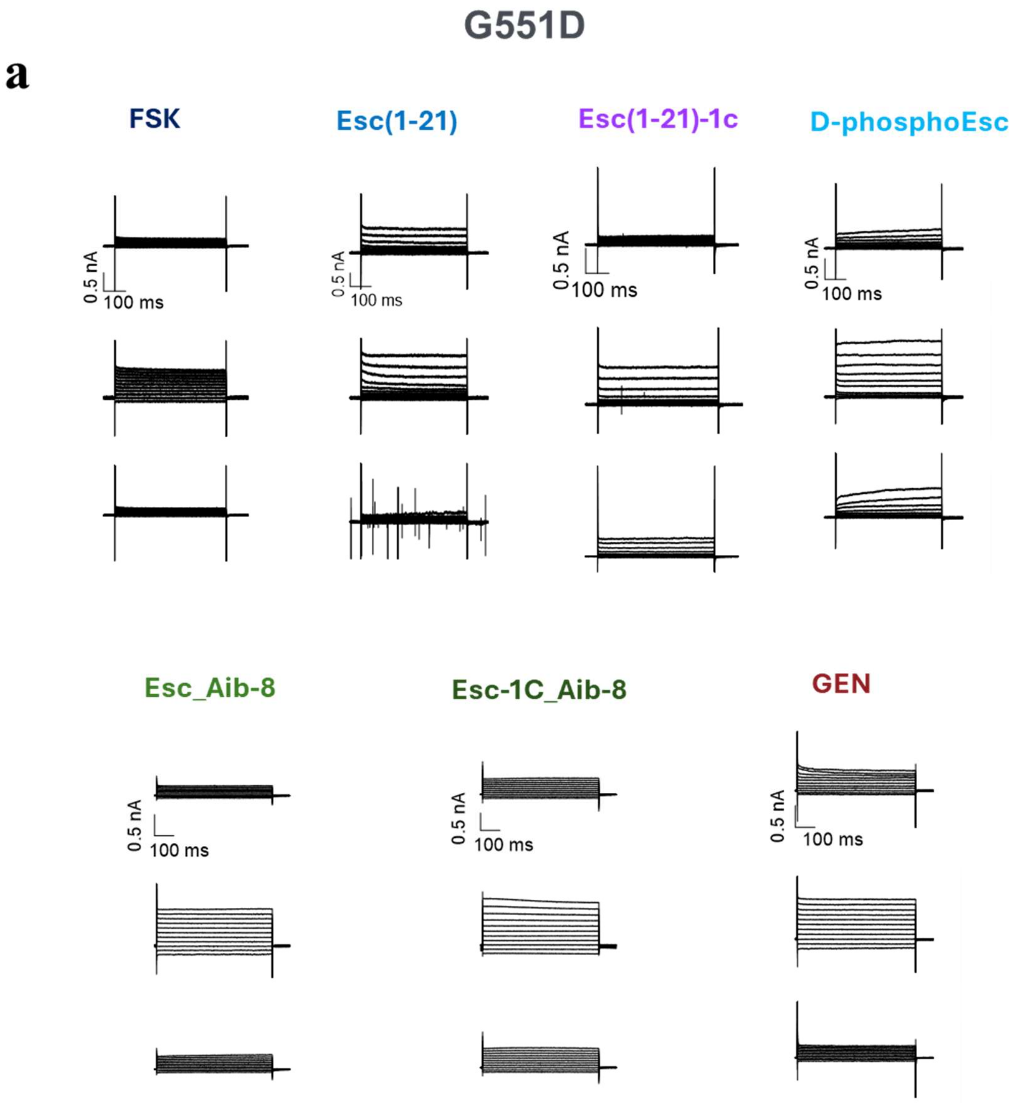

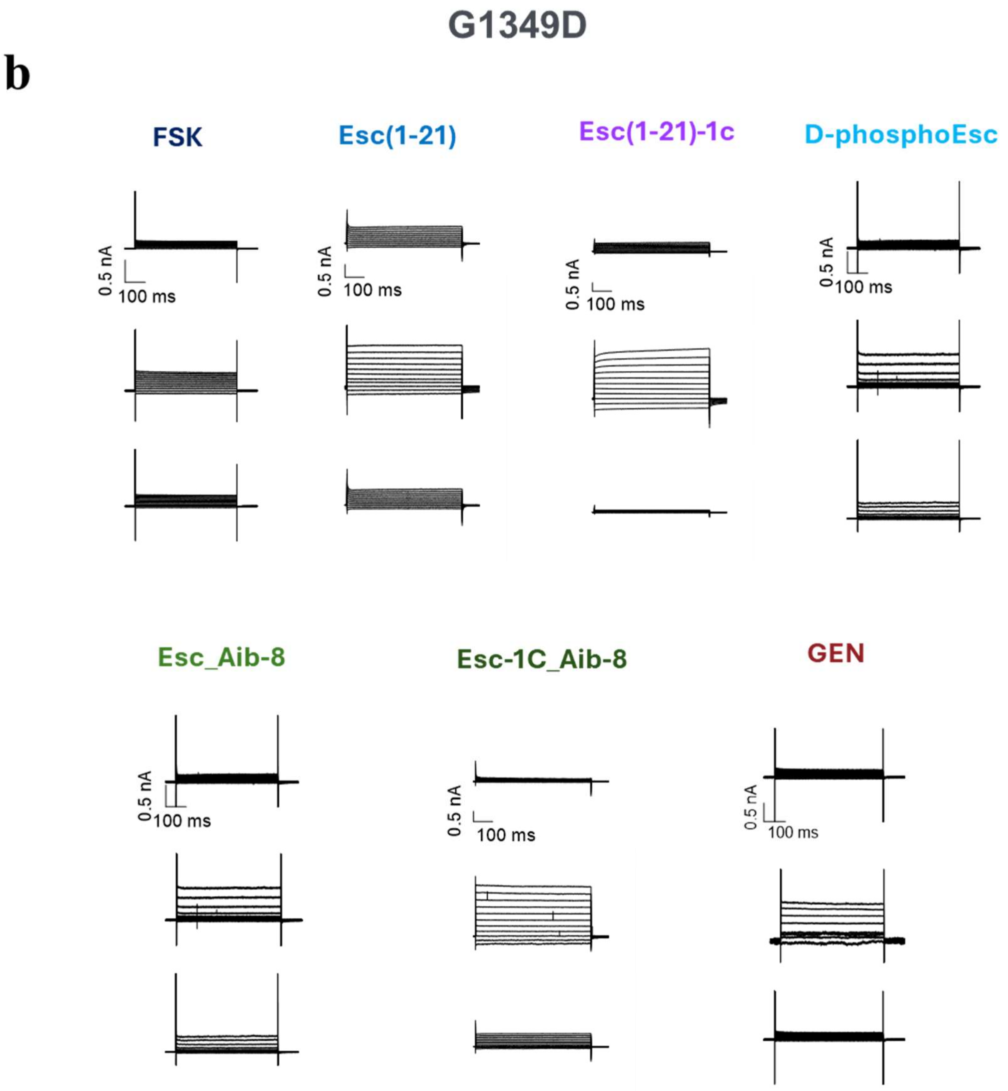

Fig. S1. Representative whole-cell membrane currents from patch-clamp experiments in G551D (a) and G1349D-FRT (b) cells. The figure shows superimposed currents elicited at membrane potentials in the range -100 to +100 mV in the control external solution (top panel), after application of 20 μM FSK alone or FSK + 10 μM peptides/GEN, as indicated (central panel) and after application of 10 μM Inhibitor 172 (bottom panel)

The effect of FSK alone or FSK plus each peptide on the intracellular calcium ions evaluated by the Fluo-4 assay in null FRT cells.

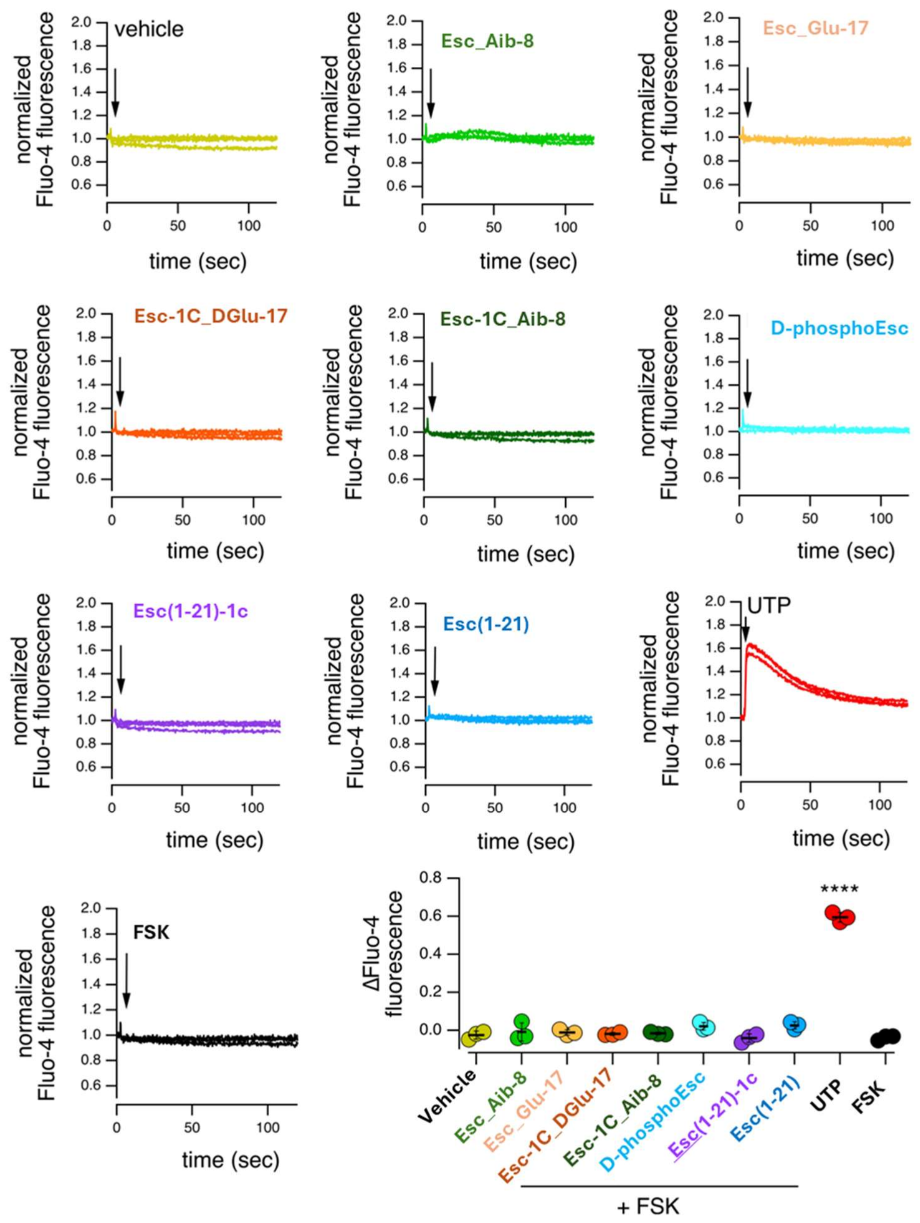

Fig. S2. Effect of peptides on intracellular calcium in the presence of FSK. Every trace in the graphs represents a single replicate for each condition and reflects calcium variation during time (measured with the calcium-sensitive fluorescent probe Fluo-4). Traces show the time course of normalized fluorescence (to the background) following addition (arrows) of vehicle (DMSO), peptides (10 µM each) plus FSK (20 µM), FSK alone (20 µM), or UTP (10 µM). The scatter dot plot on the bottom summarizes all the experiments and shows the maximal fluorescence change for each stimulus. **** p<0.0001 *versus* vehicle
